# Polygenic Scores and Mood Disorder Onsets in the Context of Family History and Early Psychopathology

**DOI:** 10.1001/jamanetworkopen.2025.5331

**Published:** 2025-04-16

**Authors:** Kathryn Freeman, Alyson Zwicker, Janice M. Fullerton, Danella M. Hafeman, Neeltje E. M. van Haren, John Merranko, Benjamin I. Goldstein, Emma K. Stapp, Elena de la Serna, Dolores Moreno, Gisela Sugranyes, Sergi Mas, Gloria Roberts, Claudio Toma, Peter R. Schofield, Howard J. Edenberg, Holly C. Wilcox, Melvin G. McInnis, Lukas Propper, Barbara Pavlova, Samuel A. Stewart, Eileen M. Denovan-Wright, Guy A. Rouleau, Josefina Castro-Fornieles, Manon H. J. Hillegers, Boris Birmaher, Philip B. Mitchell, Martin Alda, John I. Nurnberger, Rudolf Uher

**Affiliations:** 1 Department of Medical Neuroscience, Dalhousie University, Halifax, Nova Scotia, Canada; 2 Nova Scotia Health Authority, Halifax, Nova Scotia, Canada; 3 Department of Psychiatry, Dalhousie University, Halifax, Nova Scotia, Canada; 4 Dalhousie Medicine New Brunswick, St John, New Brunswick, Canada; 5 Neuroscience Research Australia, Randwick, New South Wales, Australia; 6 School of Biomedical Sciences, Faculty of Medicine & Health, University of New South Wales, Sydney, New South Wales, Australia; 7 Western Psychiatric Hospital, University of Pittsburgh School of Medicine, Pittsburgh, Pennsylvania; 8 Department of Child and Adolescent Psychiatry/Psychology, Erasmus University Medical Center, Sophia Children’s Hospital, Rotterdam, the Netherlands; 9 Department of Psychiatry, University Medical Center Utrecht Brain Center, Utrecht, the Netherlands; 10 Centre for Addiction and Mental Health, Faculty of Medicine, University of Toronto, Toronto, Ontario, Canada; 11 Milken Institute School of Public Health, George Washington University, Washington, District of Columbia; 12 Fundacio Clínic per la Recerca Biomedica, Institut d'Investigacions Biomèdiques d'August Pi i Sunye, Barcelona, Spain; 13 Centro de Investigación Biomédica en Red de Salud Mental, Madrid, Spain; 14 Department of Child and Adolescent Psychiatry and Psychology, 2021 SGR 01319, Hospital Clinic of Barcelona, Barcelona, Spain; 15 Department of Child and Adolescent Psychiatry, Hospital General Universitario Gregorio Marañón, Madrid, Spain; 16 Department of Clinical Foundations, Universitat de Barcelona, Barcelona, Spain; 17 Discipline of Psychiatry and Mental Health, School of Clinical Medicine, University of New South Wales, Randwick, New South Wales, Australia; 18 Centro de Biología Molecular “Severo Ochoa”, Universidad Autónoma de Madrid, Consejo Superior de Investigaciones Científicas, Madrid, Spain; 19 Department of Biochemistry and Molecular Biology, Indiana University, Indianapolis; 20 Johns Hopkins Bloomberg School of Public Health, Baltimore, Maryland; 21 Johns Hopkins School of Medicine, Baltimore, Maryland; 22 Department of Psychiatry, University of Michigan, Ann Arbor; 23 IWK Health Centre, Halifax, Nova Scotia, Canada; 24 Department of Community Health and Epidemiology, Dalhousie University, Halifax, Nova Scotia, Canada; 25 Department of Pharmacology, Dalhousie University, Halifax, Nova Scotia, Canada; 26 Montreal Neurological Institute and Department of Neurology, McGill University, Montreal, Quebec, Canada; 27 Department of Medicine, Neurosciences Institute, University of Barcelona, Barcelona, Spain; 28 Department of Psychiatry, Indiana University School of Medicine, Indianapolis; 29 Stark Neurosciences Research Institute, Indiana University School of Medicine, Indianapolis

## Abstract

**Importance:**

Bipolar disorder (BD) and major depressive disorder (MDD) aggregate within families, with risk often first manifesting as early psychopathology, including attention-deficit/hyperactivity disorder (ADHD) and anxiety disorders.

**Objective:**

To determine whether polygenic scores (PGS) are associated with mood disorder onset independent of familial high risk for BD (FHR-BD) and early psychopathology.

**Design, Setting, and Participants:**

This cohort study used data from 7 prospective cohorts enriched in FHR-BD from Australia, Canada, the Netherlands, Spain, and the US. Participants with FHR-BD, defined as having at least 1 first-degree relative with BD, were compared with participants without FHR for any mood disorder. Participants were repeatedly assessed with variable follow-up intervals from July 1992 to July 2023. Data were analyzed from August 2023 to August 2024.

**Exposures:**

PGS indexed genetic liability for MDD, BD, anxiety, neuroticism, subjective well-being, ADHD, self-regulation, and addiction risk factor. Semistructured diagnostic interviews with relatives established FHR-BD. ADHD or anxiety disorder diagnoses before mood disorder onset constituted early psychopathology.

**Main Outcomes and Measures:**

The outcome of interest, mood disorder onset, was defined as a consensus-confirmed new diagnosis of MDD or BD. Cox regression examined associations of PGS, FHR-BD, ADHD, and anxiety with mood disorder onset. Kaplan-Meier curves and log-rank tests evaluated the probability of onset by PGS quartile and familial risk status.

**Results:**

A total of 1064 participants (546 [51.3%] female; mean [SD] age at last assessment, 21.7 [5.1] years), including 660 with FHR-BD and 404 without FHR for any mood disorder, were repeatedly assessed for mental disorders. A total of 399 mood disorder onsets occurred over a variable mean (SD) follow-up interval of 6.3 (5.7) years. Multiple PGS were associated with onset after correcting for FHR-BD and early psychopathology, including PGS for ADHD (hazard ratio [HR], 1.19; 95% CI, 1.06-1.34), self-regulation (HR, 1.19; 95% CI, 1.06-1.34), neuroticism (HR, 1.18; 95% CI, 1.06-1.32), MDD (HR, 1.17; 95% CI, 1.04-1.31), addiction risk factor (HR, 1.16; 95% CI, 1.04-1.30), anxiety (HR, 1.15; 95% CI, 1.02-1.28), BD (HR, 1.14; 95% CI, 1.02-1.28), and subjective well-being (HR, 0.89; 95% CI, 0.79-0.99). High PGS for addiction risk factor, anxiety, BD, and MDD were associated with increased probability of onset in the control group. High PGS for ADHD and self-regulation increased rates of onset among participants with FHR-BD. PGS for self-regulation, ADHD, and addiction risk factors showed stronger associations with onsets of BD than MDD.

**Conclusions and Relevance:**

In this cohort study, multiple PGS were associated with mood disorder onset independent of family history of BD and premorbid diagnoses of ADHD or anxiety. The association between PGS and mood disorder risk varied depending on family history status.

## Key Points

**Question:**

Are polygenic scores associated with mood disorder onset above family history of bipolar disorder and early psychopathology, defined as diagnosis of attention-deficit/hyperactivity disorder or anxiety before mood disorder onset?

**Findings:**

In this cohort study of 1064 participants from 7 international cohorts, 8 psychopathology-related polygenic scores were associated with mood disorder onset. Polygenic score associations differed depending on family history and were partly diagnosis-specific to bipolar disorder.

**Meaning:**

These findings suggest that polygenic scores are uniquely associated with mood disorder onset above established predictors family history and early psychopathology.

## Teaser

This cohort study uses data from 7 international cohorts enriched for familial high risk for bipolar disorder to examine whether polygenic scores (PGS) are associated with increased risk of mood disorder compared with familial high-risk for bipolar disorder and early psychopathology.

## Introduction

Mood disorders are among the most common and disabling health conditions.^1,2^ Recognizing individuals with elevated risk for mood disorders, such as bipolar disorder (BD) and major depressive disorder (MDD), creates opportunities for early intervention.^3^

Mood disorders run in families, often leading to psychopathology in the next generation. Parental diagnosis of BD increases the lifetime risk of both MDD and BD.^4^ Children with a family history of BD also experience increased odds of developing attention-deficit/hyperactivity disorder (ADHD) and anxiety.^5^

The single most reliable risk factor associated with mood disorder onset is family history of these disorders.^6^ Another potent risk factor associated with mood disorders is early psychopathology, especially childhood-onset disorders, such as ADHD and anxiety.^7,8,9^ Despite incorporating family history and early psychopathology, the current approach still falls short of clinically meaningful accuracy when estimating onsets of mood disorders.

Polygenic scores (PGS) use risk alleles from a genome-wide association study (GWAS) to assign a numeric genetic risk score for certain traits based on individuals’ genotypes.^10^ GWAS-derived effect size estimates weight single nucleotide variation genotypes that sum to create PGS. Recent GWAS have identified loci associated with psychiatric disorders, including MDD,^11^ BD,^12^ ADHD,^13^ and anxiety.^14^ Additionally, GWAS identified loci associated with behavioral traits relevant to mood disorders, including self-regulation,^15,16^ neuroticism,^17^ addiction risk factor,^18^ and subjective well-being.^19^ However, many of these PGS remain untested as risk factors for new mood disorder onset.

PGS for neuroticism and subjective well-being were associated with risk of a major mood or psychotic disorder independently of family history of such disorders.^20^ The lack of specificity in this association prompts inquiry into whether more precise factors may hold particular relevance to a certain disorder. Moreover, it raises the question of whether PGS for broad constructs, including neuroticism, are important after accounting for family history of a specific disorder, such as BD. A comprehensive meta-analysis of antecedents identified ADHD and subthreshold hypomanic symptoms as specific antecedents to BD.^21,22^ This encourages investigation to determine whether genetic predisposition to ADHD or mania holds distinct value in the context of family history of BD. However, due to the limited scale of previous diagnosis-specific studies, predominant focus on testing PGS for BD,^23^ and rapid development of PGS methods, it remains unclear whether a broader palette of psychopathology-related PGS are associated with mood disorder onsets above family history of BD and early psychopathology. It is also unknown whether PGS are associated with mood disorder onsets similarly in those with or without familial risk for BD. Our objective was to assess whether PGS are associated with mood disorder onset over early psychopathology and family history of BD. Additionally, we explored the impact of family high-risk (FHR) status on PGS associations and the relative specificity of the association with BD compared with any mood disorder onset.

## Methods

This cohort study was approved by the Nova Scotia Health Authority Research Ethics Board and used data from 7 cohort studies from Australia,^24^ Canada,^25,26^ the Netherlands,^27^ Spain,^28^ and the United States.^29,30^ Each study was approved by its local institutional review board, and all participants or their parents/guardians provided written informed consent. Reporting for this study adhered to the Strengthening the Reporting of Observational Studies in Epidemiology (STROBE) reporting guideline.

### Participants

The participants were a subset of those included in a previous analysis, selected for FHR-BD or no FHR for any mood disorder, and with additional follow-up data.^20^ The 7 included cohorts: the Bipolar and Schizophrenia Young Offspring Study by De La Serna et al,^28^ Families Overcoming Risks and Building Opportunities for Well-being study by Uher et al,^25^ Dutch Bipolar and Schizophrenia Offspring Study by Van Haren et al,^27^ Bipolar High-Risk Project by Nurnberger et al,^29^ Sydney Bipolar Kids and Sibs Study by Roberts et al,^24^ Maritime Bipolar Family Study by Cruceanu et al,^26^ and Pittsburgh Bipolar Offspring Study by Birmaher et al.^30^ Participants aged 2 to 31 years at enrollment were assessed prospectively. Recruitment methods varied among cohorts, primarily recruiting participants with a FHR-BD through mental health services or research studies and sourcing control participants through advertisement (eMethods 1 in Supplement 1).

Family history of BD was established using semistructured diagnostic interviews with biological first-degree relatives (eMethods 1 in Supplement 1). We considered participants to be at familial high-risk for BD (FHR-BD) if 1 or multiple first-degree relatives met diagnostic criteria for BD-I or BD-II, as described in eMethods 1 in Supplement 1. Control participants did not have a first-degree relative with any mood disorder. Assessors conducted diagnostic interviews with participants at regular intervals. Clinicians blind to family psychopathology confirmed participant diagnosis in consensus meetings. We defined mood disorder onset as a confirmed diagnosis of MDD, BD-I, or BD-II. We included ADHD and anxiety diagnoses as early psychopathology risk factors if onsets occurred before any mood disorder.

### Genotype Preparation and Principal Component Analysis

Cohorts underwent separate genotyping (eMethods 1 in Supplement 1). Genotype quality control and preparation followed the same protocol in each cohort (eMethods 2 in Supplement 1). EIGENSOFT version 8.0.0 smartpca inferred the top 10 eigenvectors from the oldest offspring in each family to avoid the introduction of population substructure.^31,32^ We then projected all participants onto the top 10 generated eigenvectors. We excluded 305 principal component analysis outliers that exceeded 4 SDs from the sample mean more than 5 removal iterations. We did not exclude any participants due to self-reported race or ethnicity. Ancestry was inferred through genomic methods to identify exclusions within racial and ethnic groups (eTable 1 in Supplement 1).^33,34^

### Polygenic Scoring

We selected GWAS for MDD,^11^ BD,^12^ anxiety,^14^ neuroticism,^17^ subjective well-being,^19^ ADHD,^13^ self-regulation,^15,16^ addiction risk factor,^18^ and height^35^ based on conceptual considerations and availability of summary statistics from well-powered studies (eTable 2 in Supplement 1). GWAS summary statistics underwent quality control to correct for misspecifications that impact the power of PGS.^36^ PGS risk prediction methods that model genetic architecture more formally, such as LDpred2-auto,^37^ outperform traditional clumping and thresholding methods and PRS-CS-auto.^38^ LDpred2-auto computed PGS using GWAS summary statistics, the 1 444 196 HapMap3+ variants linkage disequilibrium reference panel^39^ and corresponding linkage disequilibrium matrices with independent blocks.^40^ We determined the posterior effect size in LDpred2-auto using 30 initial values evenly distributed between 1 × 10^−4^ and 0.2 for the proportion of causal variants. We standardized PGS within cohorts and isolated the unique contribution of PGS through regression, removing the impact of population structure along the top 10 principal components using umx-4.16.0 (eFigure 1 in Supplement 1).^41^

### Statistical Analysis

After testing the proportional hazard assumptions with the Schoenfeld residuals test (eFigure 2 in Supplement 1), we fitted mixed-effects survival analyses using coxme-2.2-18.1,^42^ timereg-2.0.5,^43^ survival-3.5-7,^44^ and dynpred-0.1.2.^45^ Cox proportional hazard regression estimated the association between PGS, early psychopathology (premorbid ADHD and anxiety), FHR-BD and mood disorder onset as hazard ratios (HRs) and their 95% CIs. We used participants’ chronological age as survival time, with end points defined as mood disorder onset or censoring at the age of the last assessment. Time-to-event models, including Cox regression and Aalen additive hazards models, that use chronological age as survival time incorporate age censoring into the analysis likelihood function, removing the impact of participant age differences.^46^ We also included self-reported sex at birth and follow-up duration as baseline covariates in all models. We used family identifier as a random effect to model the nonindependence of observations among relatives. We performed 4 Cox regression analyses to estimate the association of each PGS with mood disorder onset: (1) PGS base model (ie, with only sex and follow-up duration as covariates), (2) PGS base model corrected for dichotomous FHR-BD, (3) PGS base model corrected for early psychopathology (premorbid ADHD and anxiety), and (4) PGS base model corrected for FHR-BD and early psychopathology. We used PGS for height as a negative control. We corrected for multiple testing using false discovery rate (FDR) across all tests (n = 32; *q* = .05). We used 10-fold cross-validated *C* statistics to evaluate survival models using nonredundant PGS with less than 0.3 genetic correlation (eFigure 3 in Supplement 1). We used perturbation-resampling in survc1-1.0-3^47^ to generate 95% CIs over 100 iterations. The inferences for the differences in *C* statistics between 2 competing models were evaluated through FDR-corrected *P* values.^47^ *P* values were 2-sided, and statistical significance was set at *P* ≤ .05.

We drew Kaplan-Meier curves for FHR-BD and control participants in each PGS’ lower and upper quartiles. FDR-corrected log-rank tests determined differences between survival curves (n = 6 × 8; *q* = .05). To examine the association of each PGS specifically for BD onset, we ran sensitivity analyses excluding individuals with mood disorders other than BD (FDR: n = 16; *q* = .05).

All PGS associations and global model tests satisfied the assumption of proportional hazards except models containing PGS for self-regulation (eMethods 3, eFigure 4, and eFigure 5 in Supplement 1). Data were analyzed using R software version 4.3.0 (R Project for Statistical Computing) from August 2023 to August 2024.

## Results

### Descriptive Statistics

We analyzed data on 1064 participants (546 [51.3%] female; mean [SD] age at last assessment, 21.7 [5.1] years) from 705 families, including 660 participants with FHR-BD (62.0%) and 404 participants (38.0%) without FHR of any mood disorder. Participants were followed-up longitudinally over a mean (SD) of 6.3 (5.7) years with variable follow-up intervals (median [range] follow-up duration, 3 [1-19] years). Over 6747 person-years of follow-up, we captured 399 mood disorder onsets, including 295 MDD and 104 BD, of which 314 occurred in participants with FHR-BD. The Table and eFigure 6 in Supplement 1 describe the study participants and the age distribution at the last assessment, stratified by cohort.

**Table.** Demographic and Clinical Descriptors of Contributing Cohorts

Characteristic	Cohort participants, No. (%)						
	BASYS (n = 32)	FORBOW (n = 75)	DBSOS (n = 59)	USAB (n = 222)	BK&S (n = 215)	MBFS (n = 113)	BIOS (n = 348)
Age, mean (SD), y							
At first assessment	14.6 (1.5)	13.4 (3.3)	14.2 (2.1)	16.5 (2.7)	20.5 (5.0)	19.8 (3.8)	10.7 (4.7)
At last assessment	16.8 (1.3)	18.5 (3.1)	18.6 (2.1)	18.6 (2.7)	22.5 (5.0)	23.0 (4.3)	24.4 (5.5)
Sex							
Male	16 (50.0)	32 (42.7)	35 (59.3)	111 (50.0)	103 (47.9)	49 (43.4)	172 (49.4)
Female	16 (50.0)	43 (57.3)	24 (40.7)	111 (50.0)	112 (52.1)	64 (56.6)	176 (50.6)
Familial risk for bipolar disorder	11 (34.4)	40 (53.3)	39 (66.1)	170 (76.6)	138 (64.2)	48 (42.5)	214 (61.5)
Diagnoses							
ADHD	5 (15.6)	26 (34.7)	14 (23.7)	10 (4.5)	5 (2.3)	5 (4.4)	77 (22.1)
Anxiety	9 (28.1)	37 (49.3)	6 (10.2)	56 (25.2)	38 (17.7)	21 (18.6)	154 (44.3)
Mood disorder	4 (12.5)	42 (56.0)	26 (44.1)	97 (43.7)	64 (29.8)	31 (27.4)	135 (38.8)
Major depressive disorder	4 (12.5)	40 (53.3)	21 (35.6)	73 (32.9)	57 (26.5)	18 (15.9)	82 (23.6)
Bipolar disorder	0	2 (2.7)	5 (8.5)	24 (10.8)	7 (3.3)	13 (11.5)	53 (15.2)

Abbreviations: ADHD, attention-deficit/hyperactive disorder; BASYS, Bipolar and Schizophrenia Young Offspring Study^28^; BIOS, Pittsburgh Bipolar Offspring Study^30^; BK&S, Sydney Bipolar Kids and Sibs Study^24^; DBSOS, Dutch Bipolar and Schizophrenia Offspring Study^27^; FORBOW, Families Overcoming Risks and Building Opportunities for Well-being study^25^; MBFS, Maritime Bipolar Family Study^26^; USAB, USA Bipolar High-Risk Project.^29^

### Associations With Mood Disorder Onset

First, we used Cox regression to test the associations of family history and early psychopathology with new onsets of mood disorders over the follow-up. FHR-BD (HR, 2.99; 95% CI, 2.30-3.89) and prior diagnoses of ADHD (HR, 3.32; 95% CI, 2.47-4.47) or anxiety (HR, 2.55; 95% CI, 2.03-3.20) were associated with significant risk for later mood disorder onset.

**Figure 1.**
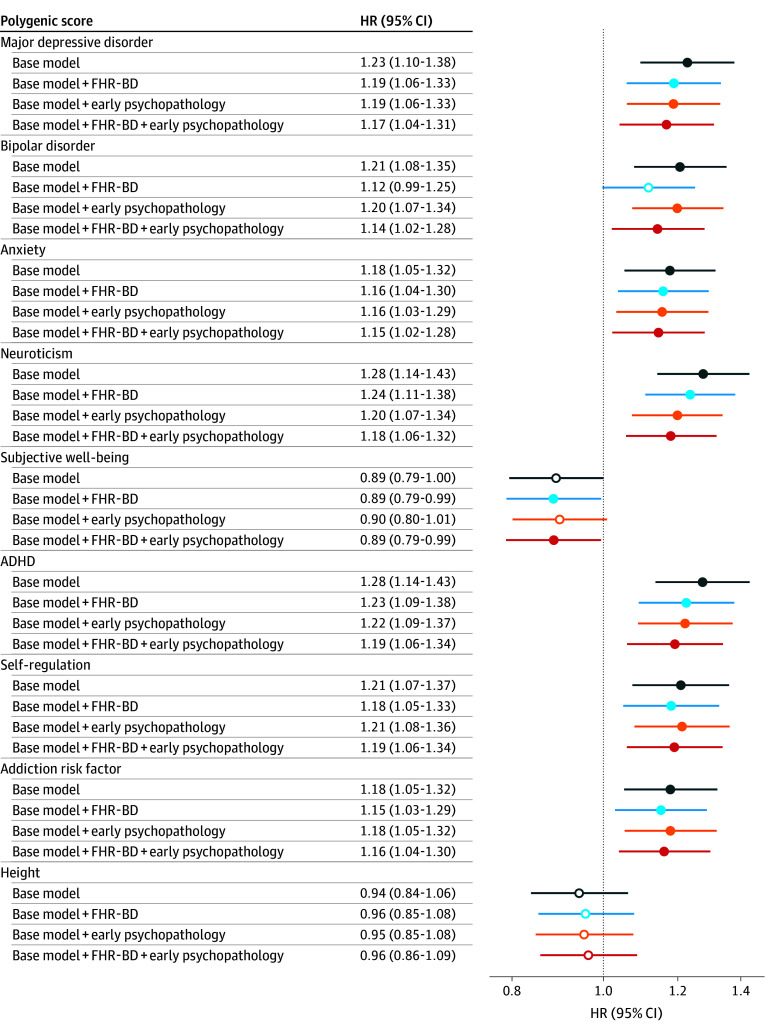
Associations of Polygenic Scores With Risk of Mood Disorder Onset ADHD indicates attention-deficit/hyperactivity disorder; FHR-BD, familial high-risk for bipolar disorder; HR, hazard ratio. Dot indicates estimate was significant; circle, estimate was not significant.

Second, we tested the association of PGS with new onsets of mood disorders (Figure 1). After correcting for FHR-BD and early psychopathology, PGS for ADHD (HR, 1.19; 95% CI, 1.06-1.34), self-regulation (HR, 1.19; 95% CI, 1.06-1.34), neuroticism (HR, 1.18; 95% CI, 1.06-1.32), MDD (HR, 1.17; 95% CI, 1.04-1.31), addiction risk factor (HR, 1.16; 95% CI, 1.04-1.30), anxiety (HR, 1.15; 95% CI, 1.02-1.28), BD (HR, 1.14; 95% CI, 1.02-1.28), and subjective well-being (HR, 0.89; 95% CI, 0.79-0.99) were independently associated with mood disorder onset. As expected, PGS for height (HR, 0.94; 95% CI, 0.84-1.06) was not associated with mood disorder onset in any models. We found significant interactions between family history and PGS for addiction risk factor (HR, 0.66; 95% CI, 0.50-0.86), anxiety (HR, 0.73; 95% CI, 0.56-0.96), and BD (HR, 0.73; 95% CI, 0.56-0.96), indicating these PGS had stronger associations with onsets in the absence of familial risk for mood disorders.

**Figure 2.**
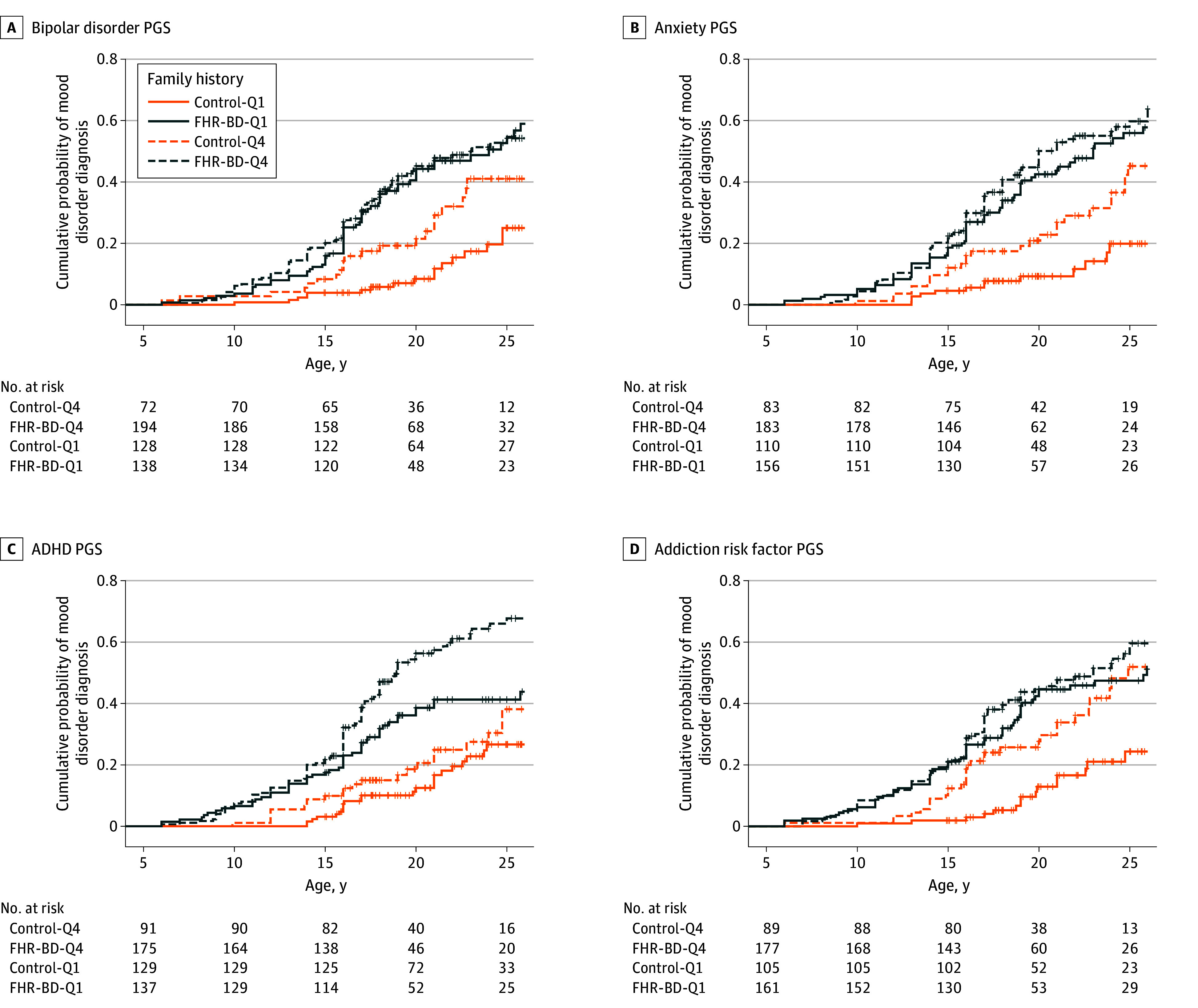
Kaplan-Meier Plots of Associations of Polygenic Score (PGS), Familial High Risk of Bipolar Disorder (FHR-BD), and Mood Disorder Onsets PGS were split into quartiles (Q) and stratified by FHR-BD: Q1 is the lowest PGS; Q4, highest PGS. ADHD indicates attention-deficit/hyperactivity disorder.

Kaplan-Meier estimates showed mood disorder onset was variably associated with PGS quartiles, depending on FHR-BD status. Log-rank tests indicated that the probability of mood disorder onset was increased in the top quartile PGS for addiction risk factor, anxiety, BD, and MDD uniquely in control participants (Figure 2). Top quartile PGS for ADHD and self-regulation were associated with elevated mood disorder onsets only among participants with FHR-BD (Figure 2). The probability of mood disorder onset was increased in the top PGS quartile for neuroticism in both FHR-BD and control participants (eFigure 7 in Supplement 1).

*C* statistics evaluated the discriminative performance of the survival model by assessing the accuracy in ranking participants based on their estimated risk of mood disorder onset (eTable 3 in Supplement 1). The difference in discriminative performance of the FHR-BD and early psychopathology model (*C* = 0.68; 95% CI, 0.65-0.71) was significantly higher than that of FHR-BD (*C* = 0.63; 95% CI, 0.61-0.66]) (*P* < .001) or early psychopathology models alone (*C* = 0.65; 95% CI, 0.62-0.67]) (*P* < .001). Models including PGS used nonredundant PGS that had less than 0.3 genetic correlation: neuroticism, ADHD, BD, and addiction risk factor (eFigure 3 in Supplement 1). The PGS model had low discriminative performance (*C* = 0.60; 95% CI, 0.57-0.63). Models including 1 clinical risk factor had significant differences in discriminative performance by adding PGS: FHR-BD (*C* = 0.65; 95% CI, 0.63-0.68]) (*P* = .04) and early psychopathology (*C* = 0.67; 95% CI, 0.64-0.70]) (*P* = .04). Adding PGS to the model with FHR-BD and early psychopathology did not have significant differences in discriminative performance (*C* = 0.70; 95% CI, 0.67-0.72]) (*P* = .12).

We assessed whether the performance of PGS survival models varied depending on FHR-BD status using the PGS identified in the Kaplan-Meier analysis (eTable 4 in Supplement 1). The discriminative performance of the PGS alone model was improved in participants without FHR-BD (*C* = 0.69; 95% CI, 0.63-0.75) and used PGS for addiction risk factor, anxiety, BD, MDD, and neuroticism. Adding early psychopathology to the model did not have a significant difference in discriminative performance (*C* = 0.71; 95% CI, 0.64-0.77]) (*P* = .42). In participants with FHR-BD, PGS for ADHD, addiction risk factor, and neuroticism had poor discriminative performance (*C* = 0.58; 95% CI, 0.54-0.61), but the model had significant improvements in discriminative performance by including early psychopathology (*C* = 0.65; 95% CI, 0.61-0.68) (*P* = .01).

### Associations With BD Onset

**Figure 3.**
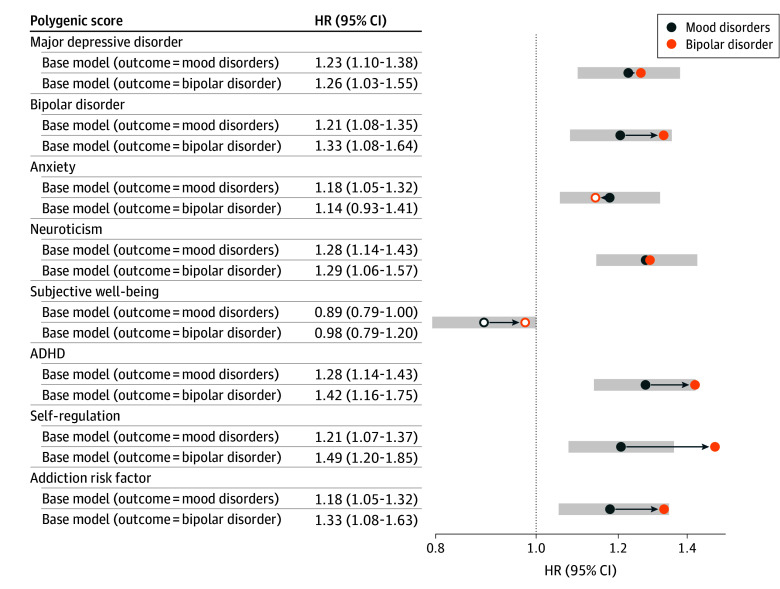
Associations of Polygenic Scores (PGS) With Bipolar Disorder Onset Compared With Mood Disorder Onsets ADHD indicates attention-deficit/hyperactivity disorder; HR, hazard ratio. Bars represent confidence intervals of mood disorder onset estimate (α = .05); dots, estimates were statistically significant; circles, estimates were not statistically significant.

The diagnosis of depression is common but always provisional, especially with a family history of BD. A diagnosis of BD is less frequent but more definite. Therefore, we completed sensitivity analyses to examine the association of each PGS specifically with the onset of BD (Figure 3). We captured 104 BD onsets, with 94 occurring in participants with FHR-BD during the follow-up period. Compared with the association with any mood disorder, PGS for self-regulation (HR, 1.49; 95% CI, 1.20-1.85), ADHD (HR, 1.42; 95% CI, 1.16-1.75), addiction risk factor (HR, 1.33; 95% CI, 1.08-1.63), and BD (HR, 1.33; 95% CI, 1.08-1.64) were associated with significantly increased hazards when forecasting BD onset. PGS for neuroticism and MDD did not change appreciably. PGS for anxiety (HR, 1.14; 95% CI, 0.93-1.41) and subjective well-being (HR, 0.98; 95% CI, 0.79-1.20) were not significantly associated with BD onset.

## Discussion

To our knowledge, this cohort study reports the most complete analysis of the role of polygenic risk in estimating the onsets of bipolar and depressive disorders in the context of family history and early psychopathology. This analysis led to 3 discoveries based on more than 1000 participants with prospective follow-ups. First, multiple PGS for psychopathology-related phenotypes were significantly associated with mood disorder onsets above the established risk factors of family history and early psychopathology. Second, PGS associations with mood disorder onsets varied depending on family history. Third, PGS associations exhibited a degree of diagnosis-specificity in BD, distinct from MDD.

We selected 8 PGS for mood disorders, internalizing and externalizing psychopathology, neurodevelopmental disorders, and factors related to substance use. Conceptual considerations and availability of summary data from well-powered GWAS guided our selection process. After multiple testing correction, all 8 psychopathology-related PGS were associated with mood disorder onsets above family history of BD and early psychopathology. These multiple associations occurred despite the PGS being mutually unrelated or only weakly correlated. These results advance prior investigations that either tested only 1 or few PGS^23,48^ or obtained less robust associations with PGS methods that do not leverage linkage disequilibrium.^20^ As expected in a sample enriched for FHR-BD, the association with PGS for BD was no longer significant after accounting for FHR-BD, indicating family history captured most of the genetic risk. Other polygenic associations remained relatively stable when controlling for FHR-BD and early psychopathology. The associations of PGS with risk of onset were stable across the adolescent and young adult age range in Aalen additive hazards models.

We further tested whether the associations with PGS differed between participants with FHR-BD and those without FHR of any mood disorders. We found that PGS for addiction risk factor, anxiety, BD, and MDD were associated with mood disorder onset more strongly in participants without FHR-BD. In contrast, PGS for ADHD and self-regulation differentiated risk most among participants with FHR-BD. PGS for neuroticism was associated mood disorder onset similarly among all participants, suggesting that polygenic risk for neuroticism is a universal risk factor for mood disorders that operates independently of family history.

While BD and depression are both highly impactful conditions, the estimated risk of BD has specific importance, as it influences clinical decisions. While the diagnosis of depressive disorders must be considered provisional (because the future potential onset of [hypo]mania cannot be precluded), the diagnosis of BD is more definite. Therefore, we examined the specificity of PGS to the onsets of BD. We found that PGS for self-regulation, ADHD, addiction risk factor, and BD showed stronger associations with onsets of BD than onsets of any mood disorder. In contrast, the associations with PGS for anxiety and subjective well-being were attenuated when specifically estimating the onset of BD. This partial specificity parallels the finding of a 2024 meta-analysis^21^ that found that ADHD was a specific risk factor associated with BD, but anxiety was a transdiagnostic factor.

Our results expanded on previous research that identified ADHD and subthreshold hypomanic symptoms as specific antecedents to BD^21^ by showing a distinct genetic predisposition through ADHD and externalizing behavior–like PGS. Our analyses confirmed previous research showing PGS for neuroticism and subjective well-being were associated with the onsets of major mood and psychotic disorders.^20^ Similarly, we showed the association with PGS for neuroticism was attenuated by family history, while PGS for subjective well-being was strengthened.

Our findings show promise to improve existing approaches for risk identification in mood disorders. Models with a *C* statistic greater than 0.7 are considered to adequately discriminate between risk profiles of individuals.^49^ Adding PGS to the early psychopathology model in those without familial risk for any mood disorder improved the estimation of mood disorder onset and allowed the *C* statistic to surpass 0.7. However, cross-validated tests of generalizable individual-level estimations did not show significant advantages over simpler models, suggesting that using PGS as clinical risk factors is not presently advisable. Despite significant findings in survival models, discriminative performance shows that the models cannot accurately rank risk of mood disorder onsets in new individuals. Therefore, our results indicate that PGS may be an adjunct to clinical and demographic information in future risk identification, if discriminative performance improves.

Although the risk of developing mood disorders is lower in individuals without family history, mood disorders still impact 6% of people with no familial risk for any mood disorder.^4^ PGS may prove valuable in the future to identify individuals at risk who may otherwise go overlooked due to negative family history. Nevertheless, the association of PGS with the probability of mood disorder onset varies depending on FHR status. Models should consider conditional associations with PGS contingent on family history. Further studies may explore the impact of family environment, which clouds associations between PGS and mood disorder onset in individuals with family history of mood disorder.

### Limitations

This study has some limitations. Although our participants had not all passed through the window of vulnerability for mood disorder onset, survival analysis is designed to handle right censoring that occurs when participants have not yet reached the milestone of interest. Participants without family history of mood disorders were younger at last assessment, which could introduce censoring bias. Despite this limitation, our analysis of time-variant effects show that results were not impacted by FHR status during the adolescent and young adult age range.

Comprehensive validation in more generalizable clinical cohorts is necessary to examine the sensitivity and specificity of using PGS in clinical settings. PGS rely on previous GWAS summary statistics to calculate scores. Currently available GWAS limits PGS strength and population diversity. Although we did not exclude participants based on self-reported race or ethnicity, racial disparities in GWAS continue to limit the generalizability of PGS. Representation across races and ethnicities in GWAS is required for equitable research in health care.

## Conclusions

In this cohort study of 1064 participants, we found that genetic liability for psychopathology-related PGS was associated with onset of mood disorders above FHR for BD and early psychopathology of premorbid ADHD and anxiety. Our results highlighted that context matters in polygenic risk where family history status impacted PGS’ ability to forecast mood disorder onset. Future work is needed to integrate PGS with clinical risk factors to achieve clinically meaningful identification of mood disorder risk.

## Notes

Supplement 1. **eMethods 1.** Cohort Recruitment, Inclusion/Exclusion Criteria, Psychopathology Assessment, and Genotyping **eMethods 2.** Genotype Quality Control and Preparation **eMethods 3.** Aalen Additive Hazards **eTable 1.** Principal Component Analysis Inclusions and Exclusions by Estimated Ancestry Grouping in Each Cohort **eTable 2.** Discovery GWAS Sample Sizes Used to Construct PGS **eTable 3.** *C* Statistics Evaluate the Performance of Survival Models With and Without Polygenic Scores **eTable 4.** *C* Statistics Evaluate the Performance of PGS Survival Models in Those With and Without Familial Risk of Bipolar Disorder **eFigure 1.** Distributions of Polygenic Scores Stratified by Familial Risk of Bipolar Disorder **eFigure 2.** Results of the Schoenfeld Residuals Test **eFigure 3.** Correlation Matrix of Polygenic Scores That Have Been Adjusted for Population Structure Along the Top 10 Principal Components **eFigure 4.** Associations of Polygenic Scores on Onsets of Mood Disorders From the Nonparametric Aalen Additive Hazard Model **eFigure 5.** Grid of Aalen Additive Hazards Models Showing the Associations of Polygenic Scores With Onsets of Mood Disorders Over Time **eFigure 6.** Distributions of Age at the Most Recent Assessment **eFigure 7.** Kaplan-Meier Plots Showing the Relationships Between Polygenic Scores and Mood Disorder Onsets
eReferences.

Supplement 2.
Data Sharing Statement
