# Supplementary material for: Polygenic Scores and Mood Disorder Onsets in the Context of Family History and Early Psychopathology
Source: JAMA Netw Open. 2025 Apr 16;8(4):e255331. doi: 10.1001/jamanetworkopen.2025.5331 (PMC12004201; doi:10.1001/jamanetworkopen.2025.5331)
Supplement: Supplement 1. — eMethods 1. Cohort Recruitment, Inclusion/Exclusion Criteria, Psychopathology Assessment, and Genotyping eMethods 2. Genotype Quality Control and Preparation eMethods 3. Aalen Additive Hazards eTable 1. Principal Component Analysis Inclusions and Exclusions by Estimated Ancestry Grouping in Each Cohort eTable 2. Discovery GWAS Sample Sizes Used to Construct PGS eTable 3. C Statistics Evaluate the Performance of Survival Models With and Without Polygenic Scores eTable 4. C Statistics Evaluate the Performance of PGS Survival Models in Those With and Without Familial Risk of Bipolar Disorder eFigure 1. Distributions of Polygenic Scores Stratified by Familial Risk of Bipolar Disorder eFigure 2. Results of the Schoenfeld Residuals Test eFigure 3. Correlation Matrix of Polygenic Scores That Have Been Adjusted for Population Structure Along the Top 10 Principal Components eFigure 4. Associations of Polygenic Scores on Onsets of Mood Disorders From the Nonparametric Aalen Additive Hazard Model eFigure 5. Grid of Aalen Additive Hazards Models Showing the Associations of Polygenic Scores With Onsets of Mood Disorders Over Time eFigure 6. Distributions of Age at the Most Recent Assessment eFigure 7. Kaplan-Meier Plots Showing the Relationships Between Polygenic Scores and Mood Disorder Onsets eReferences. [file jamanetwopen-e255331-s001.pdf]

# Supplemental Online Content

Freeman K, Zwicker A, Fullerton JM, et al. Polygenic scores and mood disorder onsets in the context of family history and early psychopathology. *JAMA Netw Open*. 2025;8(4):e255331. doi:10.1001/jamanetworkopen.2025.5331

**eMethods 1.** Cohort Recruitment, Inclusion/Exclusion Criteria, Psychopathology Assessment, and Genotyping
**eMethods 2.** Genotype Quality Control and Preparation
**eMethods 3.** Aalen Additive Hazards
**eTable 1.** Principal Component Analysis Inclusions and Exclusions by Estimated Ancestry Grouping in Each Cohort
**eTable 2.** Discovery GWAS Sample Sizes Used to Construct PGS
**eTable 3.** *C* Statistics Evaluate the Performance of Survival Models With and Without Polygenic Scores
**eTable 4.** *C* Statistics Evaluate the Performance of PGS Survival Models in Those With and Without Familial Risk of Bipolar Disorder
**eFigure 1.** Distributions of Polygenic Scores Stratified by Familial Risk of Bipolar Disorder
**eFigure 2.** Results of the Schoenfeld Residuals Test
**eFigure 3.** Correlation Matrix of Polygenic Scores That Have Been Adjusted for Population Structure Along the Top 10 Principal Components
**eFigure 4.** Associations of Polygenic Scores on Onsets of Mood Disorders From the Nonparametric Aalen Additive Hazard Model
**eFigure 5.** Grid of Aalen Additive Hazards Models Showing the Associations of Polygenic Scores With Onsets of Mood Disorders Over Time
**eFigure 6.** Distributions of Age at the Most Recent Assessment
**eFigure 7.** Kaplan-Meier Plots Showing the Relationships Between Polygenic Scores and Mood Disorder Onsets
**eReferences.**

This supplemental material has been provided by the authors to give readers additional information about their work.

© 2025 Freeman K et al. *JAMA Network Open*.

## eMethods 1: Cohorts

**1.1 - Bipolar and Schizophrenia Young Offspring Study *(BASYS)***[1]

Hospital psychiatrists recruited parents with bipolar disorder or schizophrenia. A psychiatrist established parent diagnoses through semi-structured interview using the Spanish version of the Structured Clinical Interview for DSM-IV Disorders (SCID-IV).[2] Matched controls were enrolled through advertisements in primary care and community centres.

Offspring participants between ages 6 and 17 were invited to participate in the study. We included offspring with a family history of bipolar disorder and community-matched controls without a family history of severe mental illness in this analysis. Offspring with intellectual disability, significant head injury, and current medical or neurological conditions were excluded. Child psychiatrists blind to parent diagnoses assessed offspring using the Spanish version of the Kiddie Schedule for Affective Disorders and Schizophrenia – Present and Lifetime version (K-SADS-PL),[3] self and parent report.

Genomic DNA was extracted from saliva (Oragene kit)[4] and blood samples (Roche MagNA Pure LC System). Nanodrop spectrophotometry measured sample absorbance, and DNA purity was assessed using the ratio of absorbance at 260nm and 280nm. SNPs were genotyped using the Affymetrix Axiom® Spain Biobank Array GRCh37.[5]

**1.2 - Families Overcoming Risks and Building Opportunities for Well-being study *(FORBOW)***[6]

Clinicians recruited parents with mental illness through adult mental health services in Nova Scotia, Canada. Parent diagnoses were established using semi-structured diagnostic interviews: the Schedule for Affective Disorders and Schizophrenia (SADS-IV)[7] and the Structured Clinical Interview for DSM-5 (SCID-5).[8] Diagnoses were confirmed by a psychiatrist in consensus meetings. Community-matched controls were enrolled through acquaintance referrals or school recruitment.

We conducted baseline assessments in all consenting (or assenting) biological offspring between ages 3 and 22. We included offspring with a family history of bipolar disorder and community-matched controls without a family history of severe mental illness in this analysis. Siblings were included when available. Offspring with brain injury or intellectual disability which made most assessments invalid, were excluded. Assessors blind to parent psychopathology conducted annual diagnostic interviews with the offspring participants: the K-SADS-PL,[3] self and parent report adapted for DSM-5 (< 18 years of age) or the SCID-5[8] (≥ 18 years of age). Psychiatrists blind to parent psychopathology confirmed diagnoses in consensus meetings.

We extracted genomic DNA through saliva using the Oragene kit.[4] Nanodrop spectrophotometry measured sample absorbance, and DNA purity was assessed using the ratio of absorbance at 260nm and 280nm. Single nucleotide polymorphisms (SNPs) were genotyped over five batches using Illumina's Infinium Global Screening Array v2.0 GRCh37 and v3.0 GRCh38[9] at The Centre for Applied Genomics, The Hospital for Sick Children (SickKids), Toronto, Canada.

**1.3 - USA Bipolar High-Risk Project *(USAB)***[10]

Probands (Parents or siblings) with bipolar disorder (type I), bipolar disorder (type II) with recurrent major depression, or schizoaffective disorder (bipolar type) were recruited through inpatient or outpatient adult mental services at one of four sites: Indiana University School of Medicine, Indianapolis, USA (coordinating site); University of Michigan, Ann Arbor, USA; The Johns Hopkins University School of Medicine, Baltimore, Maryland, USA; and Washington University at St Louis, St Louis, Missouri, USA. Probands were assessed for DSM-IV disorders using the Diagnostic Interview for Genetic Studies (DIGS)[11] and the Family Instrument for Genetic Studies.[12] Control families were recruited through general medicine clinics, campus advertising, and motor vehicle records.

Offspring aged 10 to 22 years were invited to participate in the study. We included offspring with a family history of bipolar disorder and community-matched controls without a family history of severe mental illness in this analysis. Assessors blind to the specific hypothesis of the study conducted diagnostic interviews with offspring participants using the K-SADS-BP,[3,10] both self- and parent-report. Two clinicians blind to parent diagnosis confirmed diagnoses in consensus meetings.

Genomic DNA was extracted from whole blood by the Rutgers University Cell and DNA Repository (New Brunswick, NJ).[13] SNP genotyping was conducted together with BK&S samples using the Illumina Infinium PsychArray BeadChip GRCh37[14] at the Mt. Sinai School of Medicine Genomics Core Facility.

© 2025 Freeman K et al. *JAMA Network Open*.

**1.4 - Dutch Bipolar and Schizophrenia Offspring Study *(DBSOS)*[15]**

Offspring of parents with bipolar disorder or schizophrenia were recruited through their psychiatrist at the University Medical Center Utrecht or mental health care centres in the Netherlands, a family members' psychiatrist or by advertisement. Parent diagnoses were verified using semi-structured diagnostic interviews: the Structured Clinical Interview for DSM-IV Axis I Disorders (SCID-I)[16] with the affected parent and the mini-Schedule for Clinical Assessment in Neuropsychiatry (mini-SCAN)[17] with their partner. If the mini-SCAN revealed indications of psychopathology, a subsequent SCID-I assessment was conducted. Control offspring were recruited through school advertisements, leisure clubs, or hospital staff.

Offspring aged 8 to 18 years were invited to participate in the study. We included offspring with a family history of bipolar disorder and community-matched controls without a family history of severe mental illness in this analysis. Offspring with severe physical illness, neurological problems (e.g., head injury or epilepsy), and IQ below 70 were excluded. Assessors conducted diagnostic interviews with offspring using the K-SADS-PL[3] (self- and parent-report) at baseline and four-year follow-up. Diagnoses were evaluated by psychiatrists and confirmed through consensus meetings.

Genomic DNA was extracted from whole blood and genotypes were obtained through the Illumina Infinium OmniExpressExome-8 Kit v1.2 on Illumina's 550K platform (GRCh37).

**1.5 - Sydney Bipolar Kids and Sibs study *(BK&S)*[18]**

Probands with bipolar disorder (type I), bipolar disorder (type II) with recurrent major depression, or schizoaffective disorder (bipolar type) were recruited through clinicians, a specialized bipolar disorder research clinic, bipolar disorder pedigree studies, mental health consumer organization and advertisements. Family history of bipolar disorder was initially assessed through the Family Interview for Genetic Studies (FIGS)[19] with at least one parent of each participant. Parents were then evaluated for DSM-IV disorders using the DIGS[11] and the Family Instrument for Genetic Studies.[12] Control participants were recruited in universities and local communities through print and electronic media. Control participants did not have a first-degree relative with either bipolar disorder (BD) I or II, recurrent major depressive disorder, schizoaffective disorder, recurrent substance abuse or any past psychiatric hospitalisation. Additionally, they did not have a second-degree relative with a history of psychosis or who had been hospitalised for a mood disorder.

Offspring aged 11 to 31 years at enrollment participated in the study. We included offspring with a family history of bipolar disorder and community-matched controls without a family history of severe mental illness in this analysis. Siblings were included when available. Assessors conducted biennial diagnostic interviews with the offspring participants: the K-SADS-BP,[3,10] self- and parent-report (< 21 years of age) or the DIGS,[11] present and lifetime diagnosis (≥ 21 years of age). Two independent raters confirmed diagnoses in consensus meetings.

Genetic Repositories Australia (Sydney, Australia) extracted genomic DNA from whole blood.[13] SNP genotyping was conducted with USAB samples using the Illumina Infinium PsychArray BeadChip GRCh37[14] at the Mt. Sinai School of Medicine Genomics Core Facility.

**1.6 - Maritime Bipolar Family Study *(MBFS)*[20]**

Families were recruited through parent or grandparent contact with mental health services in Nova Scotia, Canada. Parent diagnoses were established by semi-structured diagnostic interview using the SADS-L.[7] At least two psychiatrists confirmed diagnoses in a blind consensus meeting. Control families were recruited through local advertisements.

Offspring were between ages 10 to 29 at the first assessment. We included offspring with a family history of bipolar disorder and community-matched controls without a family history of severe mental illness in this analysis. Siblings were included when available. Psychiatrists blind to parent psychopathology confirmed diagnoses obtained through semi-structured interviews the K-SADS-PL[3] (< 18 years of age) or the Schedule for Affective Disorders and Schizophrenia – Lifetime (SADS-L)[7] and the DIGS[11] (≥ 18 years of age).

Genomic DNA was extracted from venipuncture blood samples by Dr. Guy Rouleau's laboratory at McGill University.[20] SNPs were genotyped over two batches using Illumina's Infinium Global Screening Array GRCh37[9] at the McGill Genome Centre, Montreal, Canada and The Centre for Applied Genomics, The Hospital for Sick Children (SickKids), Toronto, Canada.

**1.7 - Pittsburgh Bipolar Offspring Study *(BIOS)***[21]

Parents with bipolar disorder (type I or II) were recruited through outpatient clinics, adult bipolar disorder studies and advertisements in Pittsburgh, Pennsylvania, USA. Parent diagnoses were established by semi-structured diagnostic interview using the SCID-IV.[2] Parents with a lifetime diagnosis of schizophrenia, mania or hypomania secondary to substance use, IQ below 70, or those who were unable to cooperate during interviews were excluded. Community-matched controls were healthy or had non-bipolar disorder psychopathology. Control parents who have a spouse with bipolar disorder, first or second-degree relatives with bipolar disorder, IQ below 70, or those who were unable to cooperate during interviews were excluded.

Offspring aged 2 to 19 years at enrollment participated in the study. We included offspring with a family history of bipolar disorder and community-matched controls without a family history in this analysis. Siblings were included when available. Offspring with IQ below 70, autism spectrum disorder, a condition that interfered with evaluation, or living 200 miles away from Pittsburgh, Pennsylvania, were excluded. Assessors blind to parent psychopathology conducted diagnostic interviews with the offspring participants: K-SADS-PL,[3] both self- and parent-report. Psychiatrists blind to parent psychopathology confirmed diagnoses in consensus meetings.

Genomic DNA was extracted from salvia samples using Puregene DNA extraction kits[22] at The University of Pittsburgh Medical Center. SNPs were genotyped using Illumina's Infinium Global Screening Array GRCh37[9] at The Center for Applied Genomics at Children's Hospital of Philadelphia.

© 2025 Freeman K et al. *JAMA Network Open*.

## eMethods 2: Genotype Quality Control and Preparation

Cohorts genotyped using Genome Reference Consortium Human Build GRCh38 were transformed to GRCh37 using liftOver[23] executed in R-4.3.0[24] with package bigsnpr-1.12.2.[25] We implemented pre-imputation quality control in PLINK-1.9[26] with identical procedures across all cohorts. We retained variants with minor allele frequency (MAF) greater than 1%, genotyping call rate per SNP more than 95% and without departures from Hardy-Weinberg equilibrium ($p < 10\times10^{-10}$). We excluded participants with less than 95% genotyping rate or discordance between self-reported sex at birth and genotype-derived sex. We did not exclude any participants based on self-reported gender identity.

We imputed genotypes for cohorts separately using the Michigan Imputation Server, employing Minimac4 with Eagle v2.4 phasing and the Haplotype Reference Consortium[27] r1.1 2016 reference panel (GRCh37/hg19).[28] After imputation, we excluded variants with MAF less than 1% and r-squared values below 0.8. After merging the cohorts into a single dataset, variants with MAF greater than 1% and participants with genotype completeness above 99% were retained. Heterozygosity outliers and principal component analysis (PCA) outliers, identified through the procedure below, were excluded before PGS calculation.

Cohort genotypes were merged before imputation exclusively for PCA. Variants with MAF greater than 1% and missing rates less than 10% were retained across all pre-imputation genotypes. Before PCA, regions with high linkage disequilibrium and non-autosomal regions were excluded.

© 2025 Freeman K et al. *JAMA Network Open*.

## eMethods 3: Aalen Additive Hazards - *Probing the assumption of proportional hazards*

Most, but not all Cox proportional hazard models satisfied the assumption of hazard proportionality. Some covariates exhibited minor signs of nonproportional effects. When the proportional hazards assumption is not satisfied, Aalen additive hazards model is an appropriate alternative to Cox regression.[29] Therefore, we fit Aalen additive hazards models to explore the potential temporal influences of each covariate. We repeated the four models described within the manuscript, keeping sex and follow-up duration as baseline covariates and family identification as a frailty term. Time invariance of associations were evaluated through the Kolmogorov-Smirnov test.

Aalen Additive Hazards confirmed the results of the primary analysis. Uncorrected polygenic scores (PGS) all were associated with mood disorder onset: PGS for attention-deficit/hyperactivity disorder (ADHD) (p<0.001), neuroticism (p<0.001), self-regulation (p=0.005), major depressive disorder (MDD) (p=0.006), BD (p=0.006), anxiety (p=0.007), addiction risk factor (p=0.015), and subjective well-being (p=0.049).

After correcting for familial high risk for bipolar disorder (FHR-BD) and early psychopathology (premorbid ADHD and anxiety), PGS for neuroticism (p=0.007), ADHD (p=0.009), self-regulation (p=0.009), addiction risk factor (p=0.032), subjective well-being (p=0.037), MDD (p=0.042), BD (p=0.046), and anxiety (p=0.046) were independently associated with mood disorder onset (eFigure 4, eFigure 5 in Supplement 1).

The Kolmogorov-Smirnov test indicated that the association of most PGS were time-invariant. Uncorrected models and models corrected for both FHR-BD and early psychopathology had no time-dependent variables. All models corrected for only FHR-BD saw a time-variant association with FHR-BD which influenced onsets in the teens, whereas participants without FHR-BD exhibited indistinguishable hazards from those at risk only during the early-mid 20s. PGS for anxiety, subjective well-being, and self-regulation models corrected for only early psychopathology saw another time-variant association. Specifically, a prior anxiety diagnosis contributed additional hazard, predominantly between ages 15 and 20.

© 2025 Freeman K et al. *JAMA Network Open*.

# eTables

**3.1 - eTable 1.** Principal component analysis (PCA) inclusions and exclusions by estimated ancestry grouping[30,31] in each cohort.

| | BASYS [Participants, No.] | | FORBOW [Participants, No.] | | DBSOS [Participants, No.] | | USAB [Participants, No.] | | BK&S [Participants, No.] | | MBFS [Participants, No.] | | BIOS [Participants, No.] | |
|---|---|---|---|---|---|---|---|---|---|---|---|---|---|---|
| **Ancestry** | **Included** | **Excluded** | **Included** | **Excluded** | **Included** | **Excluded** | **Included** | **Excluded** | **Included** | **Excluded** | **Included** | **Excluded** | **Included** | **Excluded** |
| Total | 32 | 0 | 75 | 79 | 59 | 3 | 222 | 10 | 215 | 12 | 113 | 41 | 348 | 160 |
| Africa (North) | 0 | 0 | 0 | 1 | 0 | 0 | 0 | 0 | 0 | 0 | 0 | 0 | 0 | 0 |
| Africa (West) | 0 | 0 | 0 | 0 | 0 | 0 | 0 | 0 | 0 | 0 | 0 | 1 | 0 | 40 |
| Europe (East) | 0 | 0 | 1 | 0 | 0 | 0 | 11 | 5 | 12 | 1 | 3 | 0 | 87 | 9 |
| Europe (North West) | 0 | 0 | 72 | 71 | 59 | 3 | 204 | 5 | 182 | 4 | 103 | 34 | 244 | 27 |
| Europe (South West) | 32 | 0 | 1 | 0 | 0 | 0 | 5 | 0 | 12 | 2 | 6 | 1 | 17 | 11 |
| Middle East | 0 | 0 | 0 | 0 | 0 | 0 | 0 | 0 | 1 | 1 | 0 | 0 | 0 | 0 |
| South America | 0 | 0 | 0 | 0 | 0 | 0 | 0 | 0 | 0 | 2 | 0 | 0 | 0 | 0 |
| South Asia | 0 | 0 | 0 | 0 | 0 | 0 | 0 | 0 | 0 | 0 | 0 | 2 | 0 | 0 |
| Admixed | 0 | 0 | 1 | 7 | 0 | 0 | 2 | 0 | 8 | 2 | 1 | 3 | 0 | 73 |

**Cohort abbreviations:** BASYS = Bipolar and Schizophrenia Young Offspring Study, De La Serna et al,[1] 2017; FORBOW = Families Overcoming Risks and Building Opportunities for Well-being study, Uher et al,[6] 2014; DBSOS = Dutch Bipolar and Schizophrenia Offspring Study Van Haren et al,[15] 2020; USAB = USA Bipolar High-Risk Project, Nurnberger et al,[10] 2011; BK&S = Sydney Bipolar Kids and Sibs study, Roberts et al,[18] 2013; MBFS = Maritime Bipolar Family Study, Cruceanu et al,[20] 2018; BIOS = Pittsburgh Bipolar Offspring Study, Birmaher et al,[21] 2009.

© 2025 Freeman K et al. *JAMA Network Open*.

**3.2 - eTable 2.** Discovery GWAS sample sizes used to construct PGS.

| Phenotype | Sample Size (n, [cases if not continuous phenotype]) |
|---|---|
| Addiction Risk Factor[32] | 1,025,550 |
| ADHD[33] | 225,534; 38,691 cases |
| Anxiety (continuous GAD-2)[34] | 199,611 |
| Bipolar Disorder[35] | 413,466; 41,917 cases |
| Height[36] | 1,632,839 |
| Major Depressive Disorder[37] | 173,005; 59,851 cases |
| Neuroticism[38] | 390,278 |
| Self-regulation[39,40] | 1,045,957 |
| Subjective Wellbeing[41] | 204,966 |

**Discovery GWAS:** Addiction Risk Factor, Hatoum et al,[32] 2023; ADHD, Demontis et al,[33] 2023; Anxiety, Levey et al,[34] 2020; Bipolar Disorder, Mullins et al,[35] 2021; Height, Yengo et al,[36] 2022; Major Depressive Disorder, Wray et al,[37] 2018; Neuroticism, Nagel et al,[38] 2018; Self-regulation, Linnér et al,[39] 2021, Williams et al,[40] 2023; Subjective Wellbeing, Okbay et al,[41] 2016.

© 2025 Freeman K et al. *JAMA Network Open*.

## 3.3 - **eTable 3.** C-statistics evaluate the performance of survival models with and without polygenic scores.

| | PGS Alone *[c statistic, (95% CI)]* | FHR-BD *[c statistic, (95% CI)]* | Early Psychopathology *[c statistic, (95% CI)]* | FHR-BD and Early Psychopathology *[c statistic, (95% CI)]* |
|---|---|---|---|---|
| Non-Genetic Predictors Only | | 0.63 (0.61-0.66), **p<0.001** | 0.65 (0.62-0.67), **p<0.001** | 0.68 (0.65-0.71) |
| PGS | 0.60 (0.57-0.63) | 0.65 (0.63-0.68), **p=0.04** | 0.67 (0.64-0.70), **p=0.04** | 0.70 (0.67-0.72), p=0.12 |

PGS = non-redundant polygenic scores (ADHD, Neuroticism, Addiction Risk Factor, BD); significant interactions between PGS & FHR-BD were included; c > 0.7 indicates adequate discrimination between risk profiles; PGS p-values were calculated with reference to the non-genetic predictors only values, FHR-BD only and early psychopathology only p-values were calculated in reference to the FHR-BD and early psychopathology model; bolded p-values indicates a significant difference in c-index by including PGS in the model (FDR: n=5, q=0.05).

## 3.4 - **eTable 4.** C-statistics evaluate the performance of PGS survival models in those with and without familial risk of bipolar disorder.

| | *Familial Risk Status* | |
|---|---|---|
| | FHR-BD [c statistic, (95% CI)] | Control [c statistic, (95% CI)] |
| Early Psychopathology | 0.63 (0.60-0.66) | 0.63 (0.57-0.68) |
| PGS | 0.58 (0.54-0.61), p=0.45 | 0.69 (0.63-0.75), p=0.28 |
| PGS and Early Psychopathology | 0.65 (0.61-0.68), p=0.33 | **0.71** (0.64-0.77), p=0.12 |

Polygenic scores (PGS) used were determined based on Kaplan Meier Analysis (PGS FHR-BD = ADHD, Self Regulation, Neuroticism; PGS Control = Addiction Risk Factor, Anxiety, BD, MDD, Neuroticism); c > 0.7 indicates adequate discrimination between risk profiles; p-values calculated with reference to the early psychopathology values; bolded font indicates a significant improvement in c-index by including PGS in the model before FDR multiple comparison correction (FDR: n=6, q=0.05)

© 2025 Freeman K et al. *JAMA Network Open*.

## eFigures

**4.1 - eFigure 1.** Distributions of polygenic scores stratified by familial risk of bipolar disorder.

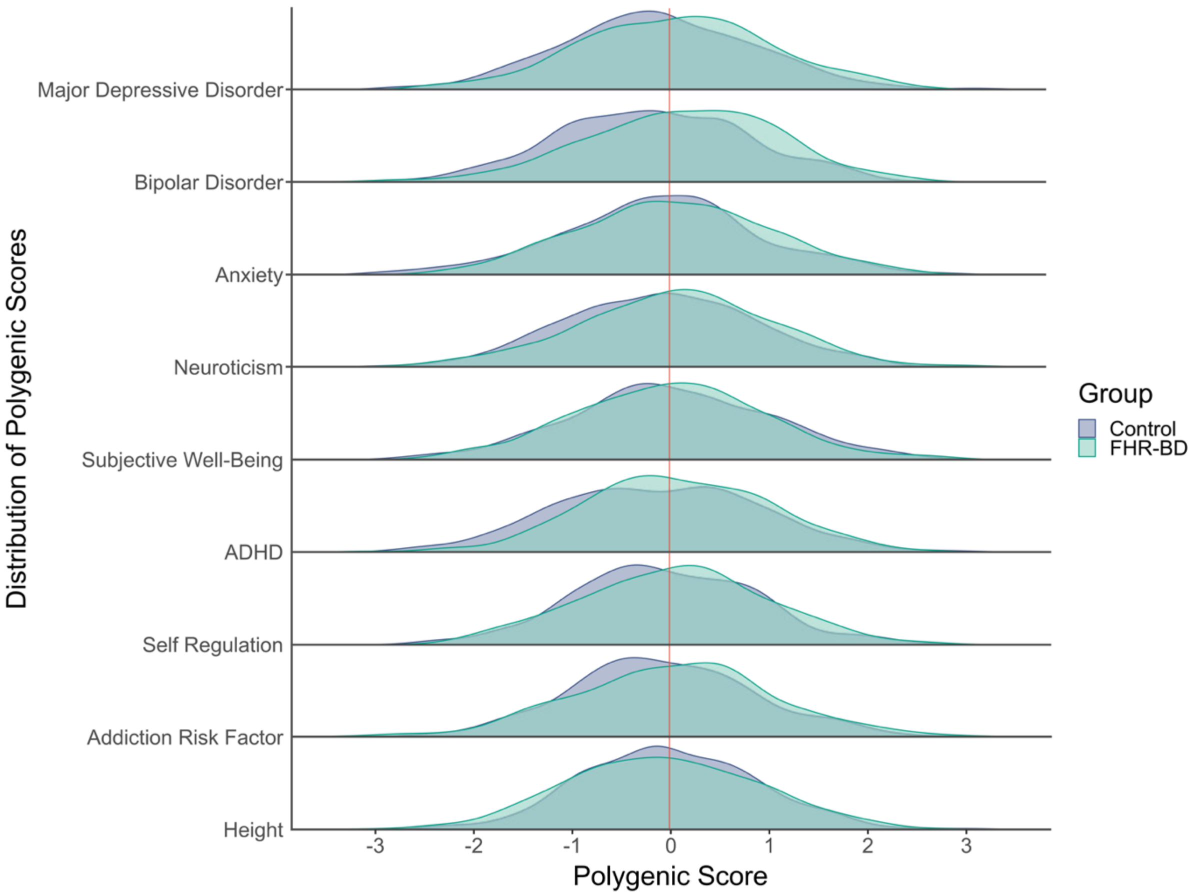


**Discovery GWAS:** Addiction Risk Factor, Hatoum et al,[32] 2023; ADHD, Demontis et al,[33] 2023; Anxiety, Levey et al,[34] 2020; Bipolar Disorder, Mullins et al,[35] 2021; Height, Yengo et al,[36] 2022; Major Depressive Disorder, Wray et al,[37] 2018; Neuroticism, Nagel et al,[38] 2018; Self-regulation, Linnér et al,[39] 2021, Williams et al,[40] 2023; Subjective Wellbeing, Okbay et al,[41] 2016.

© 2025 Freeman K et al. *JAMA Network Open*.

**4.2** - **eFigure 2.** Results of the Schoenfeld residuals test.

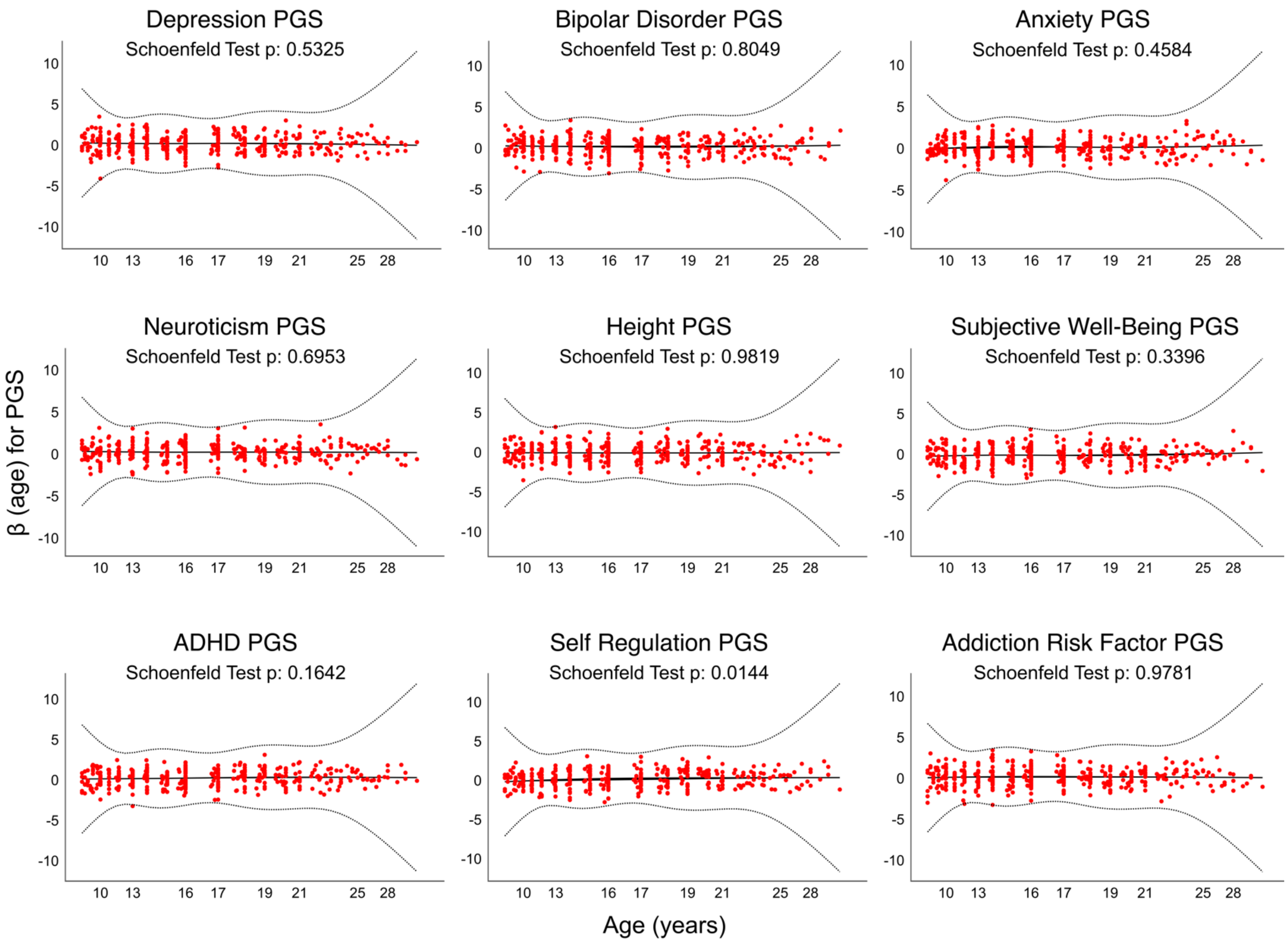


p > 0.05 indicates the proportional hazards assumption has not been violated; PGS = polygenic score. **Discovery GWAS:** Addiction Risk Factor, Hatoum et al,[32] 2023; ADHD, Demontis et al,[33] 2023; Anxiety, Levey et al,[34] 2020; Bipolar Disorder, Mullins et al,[35] 2021; Height, Yengo et al,[36] 2022; Major Depressive Disorder, Wray et al,[37] 2018; Neuroticism, Nagel et al,[38] 2018; Self-regulation, Linnér et al,[39] 2021, Williams et al,[40] 2023; Subjective Wellbeing, Okbay et al,[41] 2016.

© 2025 Freeman K et al. *JAMA Network Open*.

**4.3 - eFigure 3.** Correlation matrix of polygenic scores that have been adjusted for population structure along the top ten principal components.

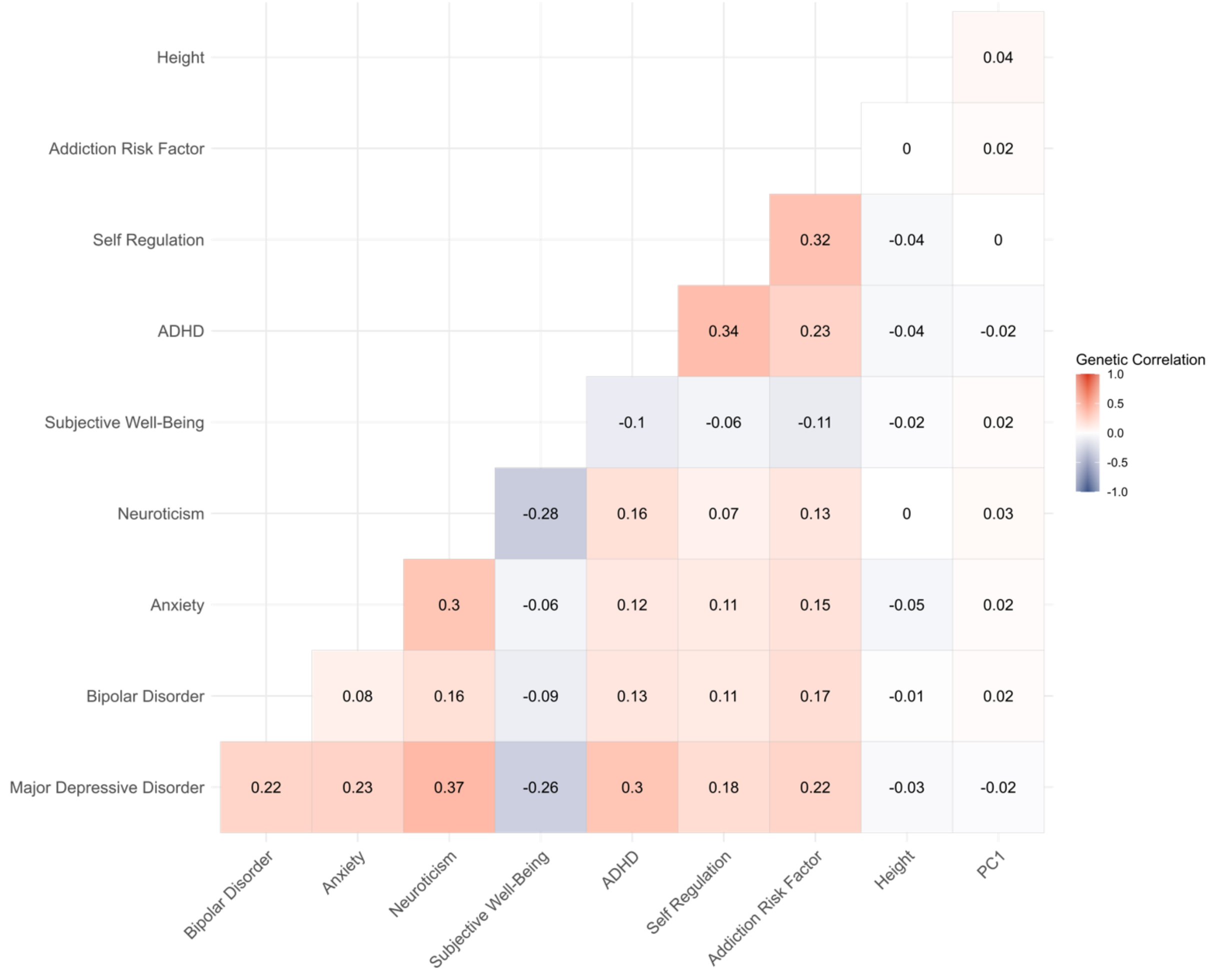


Numeric values represent Pearson correlation coefficients. **Discovery GWAS:** Addiction Risk Factor, Hatoum et al,[32] 2023; ADHD, Demontis et al,[33] 2023; Anxiety, Levey et al,[34] 2020; Bipolar Disorder, Mullins et al,[35] 2021; Height, Yengo et al,[36] 2022; Major Depressive Disorder, Wray et al,[37] 2018; Neuroticism, Nagel et al,[38] 2018; Self-regulation, Linnér et al,[39] 2021, Williams et al,[40] 2023; Subjective Wellbeing, Okbay et al,[41] 2016.

© 2025 Freeman K et al. *JAMA Network Open*.

**4.4 - eFigure 4.** Associations of polygenic scores on onsets of mood disorders from the nonparametric Aalen additive hazard model.

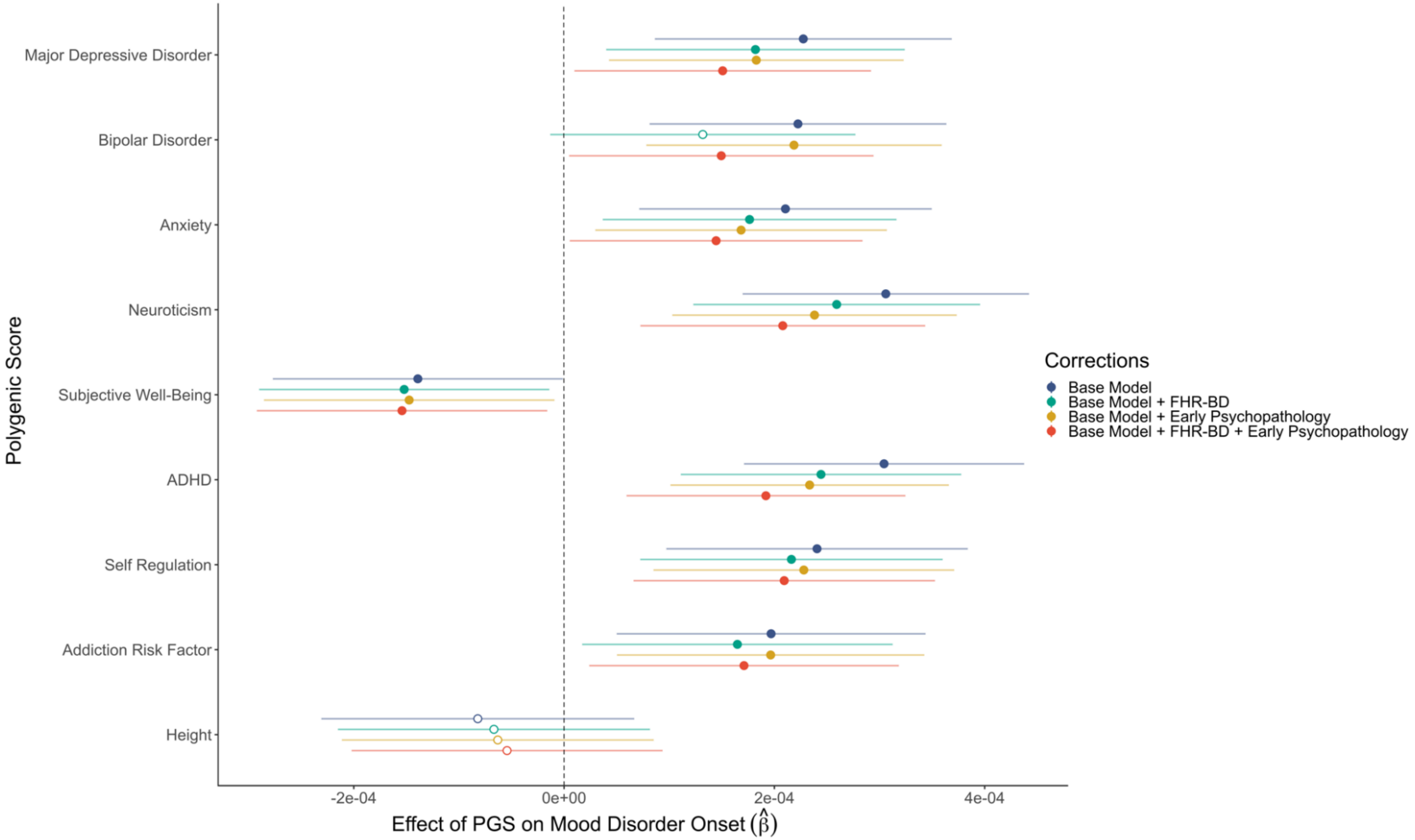


B̂ = Effect size; Lines represent confidence intervals (α = 0.05); Transparent symbols indicate estimates are not statistically significant; FHR-BD = familial high-risk for bipolar disorder; PGS = polygenic score. **Discovery GWAS:** Addiction Risk Factor, Hatoum et al,[32] 2023; ADHD, Demontis et al,[33] 2023; Anxiety, Levey et al,[34] 2020; Bipolar Disorder, Mullins et al,[35] 2021; Height, Yengo et al,[36] 2022; Major Depressive Disorder, Wray et al,[37] 2018; Neuroticism, Nagel et al,[38] 2018; Self-regulation, Linnér et al,[39] 2021, Williams et al,[40] 2023; Subjective Wellbeing, Okbay et al,[41] 2016.

© 2025 Freeman K et al. *JAMA Network Open*.

**4.5 - eFigure 5.** Grid of Aalen additive hazards models showing the associations of polygenic scores with onsets of mood disorders over time (age in years).

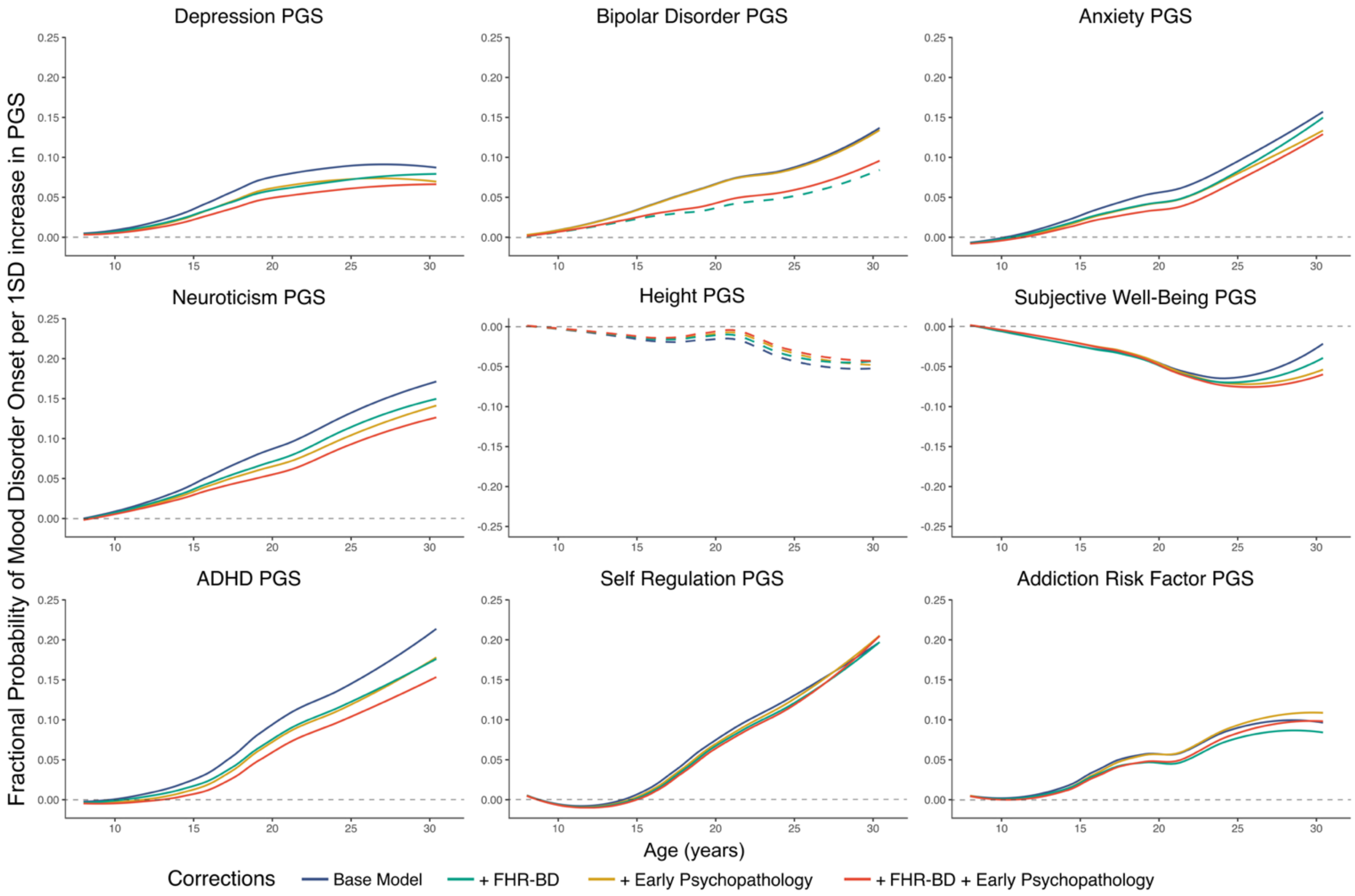


Solid lines indicate associations are statistically significant; Dotted lines indicate associations are not statistically significant; No significant time-variant associations were identified; FHR-BD = familial high-risk for bipolar disorder; PGS = polygenic score. **Discovery GWAS:** Addiction Risk Factor, Hatoum et al,[32] 2023; ADHD, Demontis et al,[33] 2023; Anxiety, Levey et al,[34] 2020; Bipolar Disorder, Mullins et al,[35] 2021; Height, Yengo et al,[36] 2022; Major Depressive Disorder, Wray et al,[37] 2018; Neuroticism, Nagel et al,[38] 2018; Self-regulation, Linnér et al,[39] 2021, Williams et al,[40] 2023; Subjective Wellbeing, Okbay et al,[41] 2016.

© 2025 Freeman K et al. *JAMA Network Open*.

**4.6 - eFigure 6.** Distributions of age at the most recent assessment.

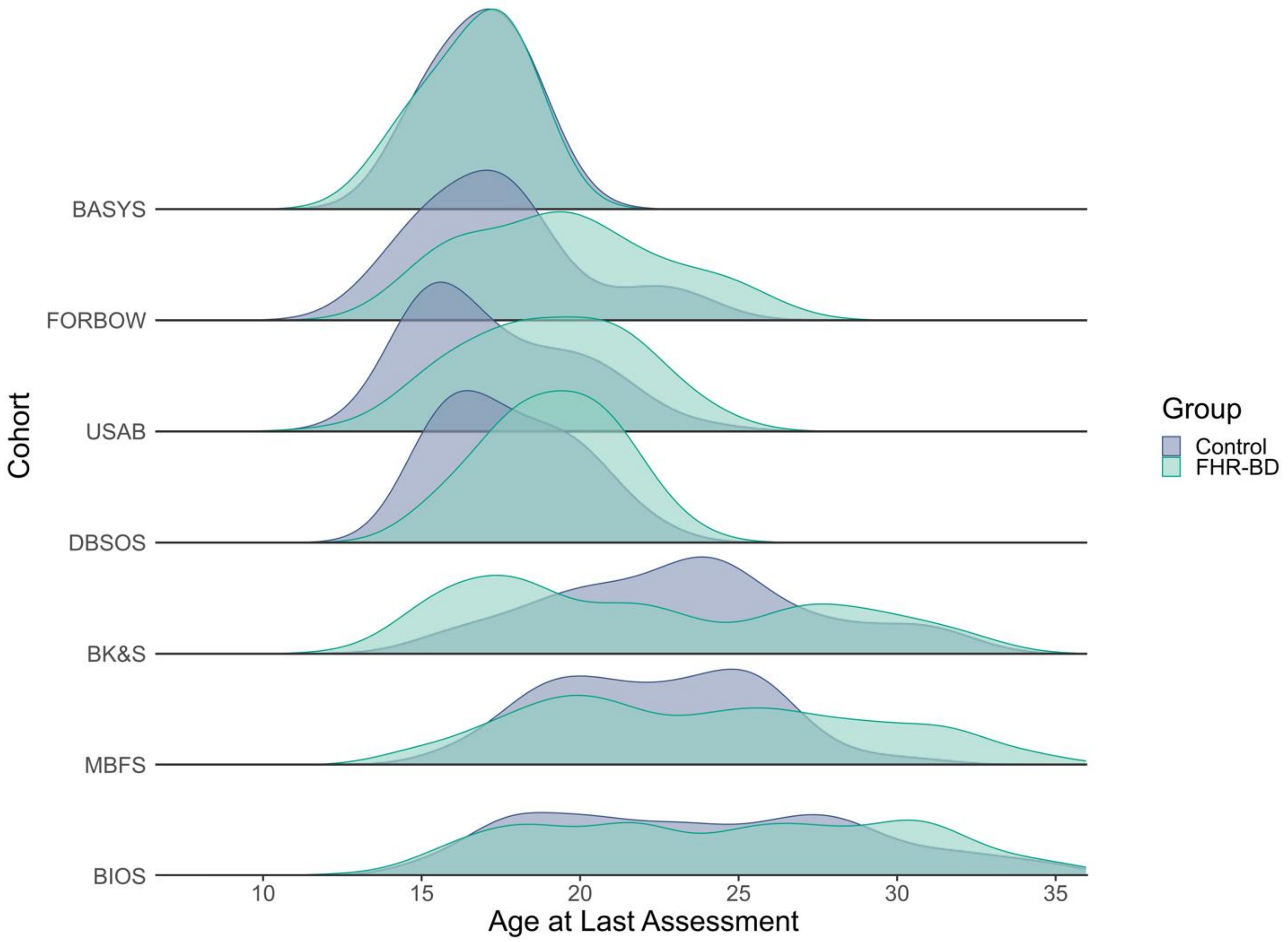


**Cohort abbreviations:** BASYS = Bipolar and Schizophrenia Young Offspring Study, De La Serna et al,[1] 2017; FORBOW = Families Overcoming Risks and Building Opportunities for Well-being study, Uher et al,[6] 2014; DBSOS = Dutch Bipolar and Schizophrenia Offspring Study Van Haren et al,[15] 2020; USAB = USA Bipolar High-Risk Project, Nurnberger et al,[10] 2011; BK&S = Sydney Bipolar Kids and Sibs study, Roberts et al,[18] 2013; MBFS = Maritime Bipolar Family Study, Cruceanu et al,[20] 2018; BIOS = Pittsburgh Bipolar Offspring Study, Birmaher et al,[21] 2009.

© 2025 Freeman K et al. *JAMA Network Open*.

**4.7** - **eFigure 7.** Kaplan-Meier plots showing the relationships between polygenic scores and mood disorder onsets. Polygenic scores were split into quartiles and stratified by family history.

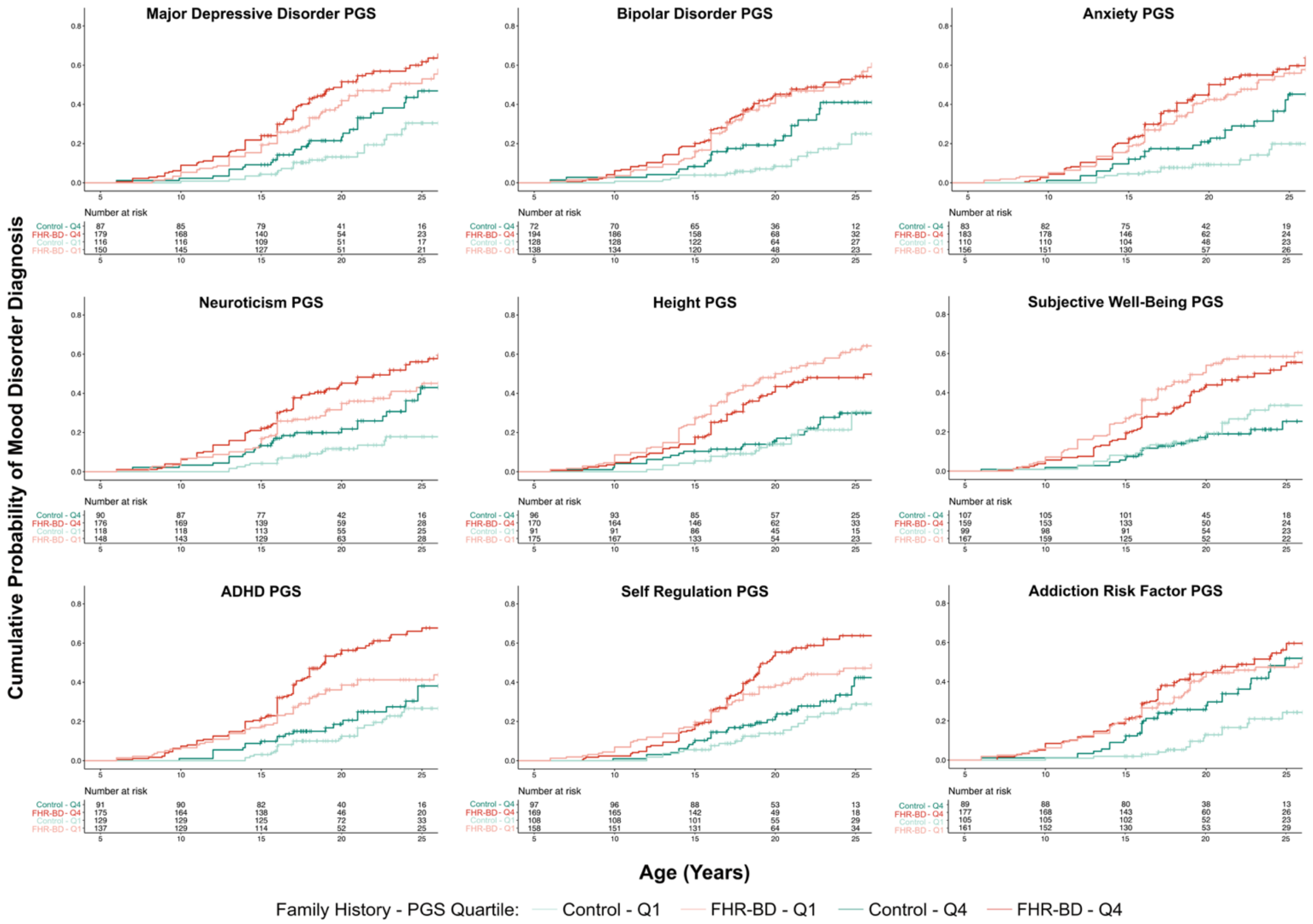


Q1 = Bottom quartile (low PGS); Q4 = Top quartile (high PGS); FHR-BD = familial high-risk for bipolar disorder; PGS = polygenic score. **Discovery GWAS:** Addiction Risk Factor, Hatoum et al,[32] 2023; ADHD, Demontis et al,[33] 2023; Anxiety, Levey et al,[34] 2020; Bipolar Disorder, Mullins et al,[35] 2021; Height, Yengo et al,[36] 2022; Major Depressive Disorder, Wray et al,[37] 2018; Neuroticism, Nagel et al,[38] 2018; Self-regulation, Linnér et al,[39] 2021, Williams et al,[40] 2023; Subjective Wellbeing, Okbay et al,[41] 2016.

© 2025 Freeman K et al. *JAMA Network Open*.

## eReferences


1. De La Serna E, Sugranyes G, Sanchez-Gistau V, Rodriguez-Toscano E, Baeza I, Vila M. Neuropsychological characteristics of child and adolescent offspring of patients with schizophrenia or bipolar disorder. *Schizophr Res*. 2017;183:110-115.

2. Spitzer RL, Williams JBW, Gibbon M, First MB. Structured clinical interview for DSM-IV (SCID). *New York: Biometrics Research*. Published online 1995.

3. Kaufman J, Birmaher B, Brent D, Rao U, Flynn C, Moreci P. Schedule for Affective Disorders and Schizophrenia for School-Age Children-Present and Lifetime Version (K-SADS-PL): Initial reliability and validity data. *J Am Acad Child Adolesc Psychiatry*. 1997;36(7):980-988.

4. DNA Genotek - Saliva DNA & RNA collection kits for research. Accessed January 24, 2024. https://dnagenotek.com/ROW/products/collection-human/collection-kits-for-research.html

5. Centro Nacional de Genotipado, Spain. Axiom Spain Biobank Array. Universidade de Santiago de Compostela. 2017. http://www.usc.es/cegen/wp-content/uploads/2019/08/COL32017-1217-Axiom-Spain-EN_FLR_FINAL.pdf

6. Uher R, Cumby J, MacKenzie LE, et al. A familial risk enriched cohort as a platform for testing early interventions to prevent severe mental illness. *BMC Psychiatry*. 2014;14:344.

7. Endicott J. A diagnostic interview: The Schedule for Affective Disorders and Schizophrenia. *Arch Gen Psychiatry*. 1978;35.

8. First MB. Structured Clinical Interview for theDSM(SCID). In: *The Encyclopedia of Clinical Psychology*. John Wiley & Sons, Inc.; 2015:1-6. doi:10.1002/9781118625392.wbecp351

9. Infinium global screening array-24 kit. Illumina. 2023. Accessed January 16, 2023. https://www.illumina.com/products/by-type/microarray-kits/infinium-global-screening.html

10. Nurnberger JI. A high-risk study of bipolar disorder: Childhood clinical phenotypes as precursors of major mood disorders. *Arch Gen Psychiatry*. 2011;68.

11. Nurnberger JI. Diagnostic Interview for Genetic Studies: Rationale, unique features, and training. *Arch Gen Psychiatry*. 1994;51.

12. FIGS Overview. Accessed January 27, 2024. https://www.nimhgenetics.org/resources/clinical-instruments/figs/overview

13. Wilcox HC, Fullerton JM, Glowinski AL, et al. Traumatic Stress Interacts With Bipolar Disorder Genetic Risk to Increase Risk for Suicide Attempts. *J Am Acad Child Adolesc Psychiatry*. 2017;56(12):1073-1080.

14. Infinium PsychArray-24 kit. Illumina. 2023. Accessed January 25, 2023. https://www.illumina.com/products/by-type/microarray-kits/infinium-psycharray.html

15. Van Haren N, Setiaman N, Koevoets M, Baalbergen H, Kahn RS, Hillegers M. Brain structure, IQ, and psychopathology in young offspring of patients with schizophrenia or bipolar disorder. *Eur Psychiatry*. 2020;63(1).

16. First MB, Gibbon M. The Structured Clinical Interview for DSM-IV Axis I Disorders (SCID-I) and the Structured Clinical Interview for DSM-IV Axis II Disorders (SCID-II). In: Hilsenroth D MJL, ed. *Comprehensive Handbook of Psychological Assessment*. Vol 2. John Wiley & Sons, Inc; 2004:134-143.


© 2025 Freeman K et al. *JAMA Network Open*.

17. Nienhuis FJ, van de Willige G, Rijnders CAT, de Jonge P, Wiersma D. Validity of a short clinical interview for psychiatric diagnosis: the mini-SCAN. *Br J Psychiatry*. 2010;196(1):64-68.

18. Roberts G, Green MJ, Breakspear M, Mccormack C, Frankland A, Wright A. Reduced inferior frontal gyrus activation during response inhibition to emotional stimuli in youth at high risk of bipolar disorder. *Biol Psychiatry*. 2013;74(1):55-61.

19. Maxwell ME. Family Interview for Genetic Studies. Bethesda, Maryland: Clinical Neurogenetics Branch, Intramural Research Program, NIMH, Bethesda, Maryland. Published online 1992. https://www.nimhgenetics.org/

20. Cruceanu C, Schmouth JF, Torres-Platas SG, Lopez JP, Ambalavanan A, Darcq E. Rare susceptibility variants for bipolar disorder suggest a role for G protein-coupled receptors. *Mol Psychiatry*. 2018;23(10):2050-2056.

21. Birmaher B, Axelson D, Monk K, Kalas C, Goldstein B, Hickey MB. Lifetime psychiatric disorders in school-aged offspring of parents with bipolar disorder: The Pittsburgh Bipolar Offspring Study. *Arch Gen Psychiatry*. 2009;66.

22. Puregene Kits. Accessed January 24, 2024. https://www.qiagen.com/us/products/discovery-and-translational-research/dna-rna-purification/dna-purification/genomic-dna/puregene-kits

23. Hinrichs AS, Karolchik D, Baertsch R, et al. The UCSC Genome Browser Database: update 2006. *Nucleic Acids Res*. 2006;34(Database issue):D590-8.

24. R Core Team. *R: A Language and Environment for Statistical Computing*. R Foundation for Statistical Computing, Vienna, Austria; 2022. https://www.R-project.org

25. Privé F, Aschard H, Ziyatdinov A, Blum MGB. Efficient analysis of large-scale genome-wide data with two R packages: bigstatsr and bigsnpr. *Bioinformatics*. 2018;34(16):2781-2787.

26. Chang CC, Chow CC, Tellier LC, Vattikuti S, Purcell SM, Lee JJ. Second-generation PLINK: rising to the challenge of larger and richer datasets. *Gigascience*. 2015;4:7.

27. McCarthy S, Das S, Kretzschmar W, et al. A reference panel of 64,976 haplotypes for genotype imputation. *Nat Genet*. 2016;48(10):1279-1283.

28. Das S, Forer L, Schönherr S, et al. Next-generation genotype imputation service and methods. *Nat Genet*. 2016;48(10):1284-1287.

29. Lefebvre F, Giorgi R. A strategy for optimal fitting of multiplicative and additive hazards regression models. *BMC Med Res Methodol*. 2021;21(1):100.

30. Privé F. Using the UK Biobank as a global reference of worldwide populations: application to measuring ancestry diversity from GWAS summary statistics. *Bioinformatics*. 2022;38(13):3477-3480.

31. Privé F, Aschard H, Carmi S, et al. Portability of 245 polygenic scores when derived from the UK Biobank and applied to 9 ancestry groups from the same cohort. *Am J Hum Genet*. 2022;109(1):12-23.

32. Hatoum AS, Colbert SMC, Johnson EC, et al. Multivariate genome-wide association meta-analysis of over 1 million subjects identifies loci underlying multiple substance use disorders. *Nature Mental Health*. 2023;1(3):210-223.

33. Demontis D, Walters GB, Athanasiadis G, et al. Genome-wide analyses of ADHD identify 27 risk loci, refine the genetic architecture and implicate several cognitive domains. *Nat Genet*. 2023;55(2):198-208.

© 2025 Freeman K et al. *JAMA Network Open*.

34. Levey DF, Gelernter J, Polimanti R, et al. Reproducible Genetic Risk Loci for Anxiety: Results From ~200,000 Participants in the Million Veteran Program. *Am J Psychiatry*. 2020;177(3):223-232.

35. Mullins N, HUNT All-In Psychiatry, Forstner AJ, et al. Genome-wide association study of more than 40,000 bipolar disorder cases provides new insights into the underlying biology. *Nat Genet*. Published online May 17, 2021. doi:10.1038/s41588-021-00857-4

36. Yengo L, Vedantam S, Marouli E, et al. A saturated map of common genetic variants associated with human height. *Nature*. 2022;610(7933):704-712.

37. Wray NR, Ripke S, Mattheisen M, et al. Genome-wide association analyses identify 44 risk variants and refine the genetic architecture of major depression. *Nat Genet*. 2018;50(5):668-681.

38. Nagel M, Jansen PR, Stringer S, et al. Meta-analysis of genome-wide association studies for neuroticism in 449,484 individuals identifies novel genetic loci and pathways. *Nat Genet*. 2018;50(7):920-927.

39. Linnér K. Multivariate analysis of 1.5 million people identifies genetic associations with traits related to self-regulation and addiction. *Nat Neurosci*. 2021;24:1367-1376.

40. Williams CM, Poore H, Tanksley PT, et al. Guidelines for evaluating the comparability of down-sampled GWAS summary statistics. *Behav Genet*. 2023;53(5-6):404-415.

41. Okbay A, Baselmans BML, De Neve JE, et al. Genetic variants associated with subjective well-being, depressive symptoms, and neuroticism identified through genome-wide analyses. *Nat Genet*. 2016;48(6):624-633.

© 2025 Freeman K et al. *JAMA Network Open*.
