# Supplementary material for: Polygenic Scores and Mood Disorder Onsets in the Context of Family History and Early Psychopathology
Source: JAMA Netw Open. 2025 Apr 16;8(4):e255331. doi: 10.1001/jamanetworkopen.2025.5331 (PMC12004201; doi:10.1001/jamanetworkopen.2025.5331)
Supplement: Supplement 2. — Data Sharing Statement [file jamanetwopen-e255331-s002.pdf]

Data Sharing Statement

Freeman. Polygenic Scores and Mood Disorder Onsets in the Context of Family History and Early Psychopathology. *JAMA Netw Open*. Published April 16, 2025.
doi:10.1001/jamanetworkopen.2025.5331

Data
Data available: No
